# Ultrafast Evolution and Loss of CRISPRs Following a Host Shift in a Novel Wildlife Pathogen, *Mycoplasma gallisepticum*

**DOI:** 10.1371/journal.pgen.1002511

**Published:** 2012-02-09

**Authors:** Nigel F. Delaney, Susan Balenger, Camille Bonneaud, Christopher J. Marx, Geoffrey E. Hill, Naola Ferguson-Noel, Peter Tsai, Allen Rodrigo, Scott V. Edwards

**Affiliations:** 1 Department of Organismic and Evolutionary Biology, Harvard University, Cambridge, Massachusetts, United States of America; 2 Department of Biological Sciences, Auburn University, Auburn, Alabama, United States of America; 3 Poultry Diagnostic and Research Center, University of Georgia, Athens, Georgia, United States of America; 4 Bioinformatics Institute, University of Auckland, Auckland, New Zealand; University of Toronto, Canada

## Abstract

Measureable rates of genome evolution are well documented in human pathogens but are less well understood in bacterial pathogens in the wild, particularly during and after host switches. *Mycoplasma gallisepticum* (MG) is a pathogenic bacterium that has evolved predominantly in poultry and recently jumped to wild house finches (*Carpodacus mexicanus*), a common North American songbird. For the first time we characterize the genome and measure rates of genome evolution in House Finch isolates of MG, as well as in poultry outgroups. Using whole-genome sequences of 12 House Finch isolates across a 13-year serial sample and an additional four newly sequenced poultry strains, we estimate a nucleotide diversity in House Finch isolates of only ∼2% of ancestral poultry strains and a nucleotide substitution rate of 0.8−1.2×10^−5^ per site per year both in poultry and in House Finches, an exceptionally fast rate rivaling some of the highest estimates reported thus far for bacteria. We also found high diversity and complete turnover of CRISPR arrays in poultry MG strains prior to the switch to the House Finch host, but after the invasion of House Finches there is progressive loss of CRISPR repeat diversity, and recruitment of novel CRISPR repeats ceases. Recent (2007) House Finch MG strains retain only ∼50% of the CRISPR repertoire founding (1994–95) strains and have lost the CRISPR–associated genes required for CRISPR function. Our results suggest that genome evolution in bacterial pathogens of wild birds can be extremely rapid and in this case is accompanied by apparent functional loss of CRISPRs.

## Author Summary

Documenting the evolutionary changes occurring in pathogens when they switch hosts is important for understanding mechanisms of adaptation and rates of evolution. We took advantage of a novel host–pathogen system involving a bacterial pathogen (*Mycoplasma gallisepticum*, or MG) and a songbird host, the House Finch, to study genome-wide changes during a host-shift. Around 1994, biologists noticed that House Finches were contracting conjunctivitis and MG from poultry was discovered to be the cause. The resulting epizootic was one of the best documented for a wildlife species, partly as a result of thousands of citizen science observers. We sequenced the genomes of 12 House Finch MG strains sampled throughout the epizootic, from 1994–2007, as well as four additional putatively ancestral poultry MG strains. Using this serial sample, we estimate a remarkably high rate of substitution, consistent with past implications that mycoplasmas are among the fastest evolving bacteria. We also find that an array of likely phage-derived sequences known as CRISPRs has degraded and ceased to recruit new repeats in the House Finch MG strains, as compared to the poultry strains in which it is diverse and rapidly evolving. This suggests that phage dynamics might be important in the dynamics of MG infection.

## Introduction

Populations of animals are under constant threat from bacterial pathogens, which can be particularly destructive following a switch to a new host or the evolution of novel virulence mechanisms. Understanding the rate and process of evolutionary change in pathogens is thus important to assessing the risks of pandemics and developing means to predict and avoid such catastrophic events. In 1994, a strain of *Mycoplasma gallisepticum* (MG) was identified as the causative agent of an emerging epizootic in House Finches, a wild songbird inhabiting Eastern North America [1]. This bacterial pathogen frequently causes disease in commercial chicken and turkey flocks, but it had never been reported in House Finches or any songbird, leading to the suggestion that the epidemic began when MG expanded its host range from poultry to this phylogenetically distant songbird. MG prevalence reached 60% in some areas, and killed an estimated 225 million finches in the first three years after detection [2]. The early detection of the epizootic allowed research and citizen-science teams to track its rapid spread throughout eastern North America in exceptional detail, making it one of the best documented wildlife pathogen outbreaks [3]–[7].

Although previous genome-wide studies have clarified rates of measurable evolution in viral pathogens [8], [9] and in bacterial populations evolving under laboratory conditions or as human pathogens [10]–[18], less is known about rates of genetic change in bacterial pathogens of non-mammalian vertebrates, particularly on short evolutionary time scales. Genome-wide and gene-specific estimates of point substitution in bacterial lineages measured over centuries [19] to millions of years [20] suggest maximum substitution rates on the order of 10^−7^ to 10^−9^ per site per year. Although recent work suggests the rate may be even faster for several bacterial species [12], [14], [19], the number of studies documenting whole-genome changes in bacteria during host switches is still small, particularly for wildlife pathogens [21], [22]. As part of ongoing surveillance, field isolates of MG obtained from infected finches were sampled at multiple time points from the start of the epidemic in 1994 to 2007, providing a genetic time series beginning immediately after the host switch, as well as an opportunity to directly measure the tempo and mode of evolution in a natural bacterial population whose genome is as yet uncharacterized.

**Figure 1:**
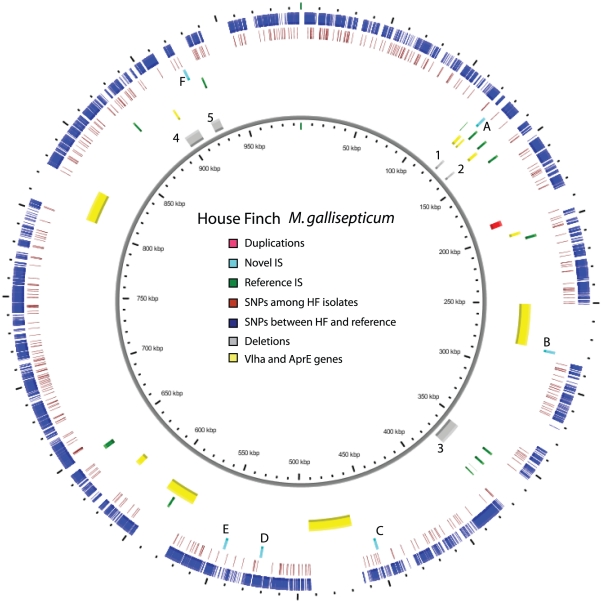
Overview of the genome of the House Finch strain of *Mycoplasma gallisepticum* summarizing variation among 12 House Finch MG isolates and comparing these to a poultry reference (0.99 Mb). Blue ticks indicate SNPs fixed within the House Finch isolates and differing from the chicken MG reference. Red ticks indicate polymorphisms among the House Finch isolates. Yellow regions are unassembled repetitive regions including VlhA and AprE genes. Grey regions indicate 4.8% of the aligned genome that is deleted in the House Finch isolates; numbers correspond to deletions detailed in Table S12. Green and light blue ticks indicate IS elements (family IS1634) in the reference genome and novel sites in the House Finch strains, respectively; letters next to novel sites correspond to insertions detailed in Table S9.

To characterize patterns of genomic change during its host switch between distantly related avian species, we sequenced whole genomes of 12 House Finch MG isolates from this 13-year time series, with four samples each from the beginning (1994–1996), middle (2001) and recent (2007) periods (Table S1). In addition, to identify putative source strains as well to determine if differences between the House Finch MG strains and the ∼1 Mb published reference R_low_ strain from chicken [23] were ancestral or derived, we sequenced four additional strains from chicken and turkey based on phylogenetic analysis of a smaller multistrain data set (Figure S1). Our sequence, SNP filtering and between-platform cross-validation protocols yielded a high quality 756,552 bp alignment encompassing 612 genes (Tables S2, S3, S4, Text S1, Figure S2), and allowed us to monitor point substitutions, genomic indels, IS element insertions, and other changes across the entire genome (Figure 1), including the entire array of clustered regularly interspaced short palindromic repeats (CRISPR) of all 17 strains (finch and poultry isolates).

## Results

### Phylogenomic diversity of House Finch and poultry MG

All House Finch MG samples were collected in the southeastern U.S. (Table S1), with an emphasis on the well studied population in Alabama [24], [25]. The population structure of Eastern House Finches before the epizootic was virtually panmictic [26], suggesting that there is likely to be little geographic structuring of MG in the east, a hypothesis that could be tested with additional data. The 12 House Finch strains from the three time periods spanned the known temporal and phylogenetic diversity of this lineage, and included strains that have been used to study host response to pathogen infection in House Finches [27]. To determine genetic diversity and phylogenetic identity of putative source populations of the House Finch MG strains, and to aid in sampling chicken and turkey strains for sequencing, we first analyzed a previously published data set [28]. Phylogenetic analysis of 1,363 bp obtained from four genomic regions for a large sample (n = 82) of MG strains suggests that turkeys rather than chickens were the source of House Finch MG and that the MG lineage colonizing House Finches first passed multiple times among chickens and turkeys (Figure S2). Although this analysis suggests frequent host switches between chickens and turkeys, which diverged 28–40 MYA [29], [30], it also suggests a single switch to the House Finch, a songbird species diverged from chickens by ∼80 MYA [31].

**Figure 2:**
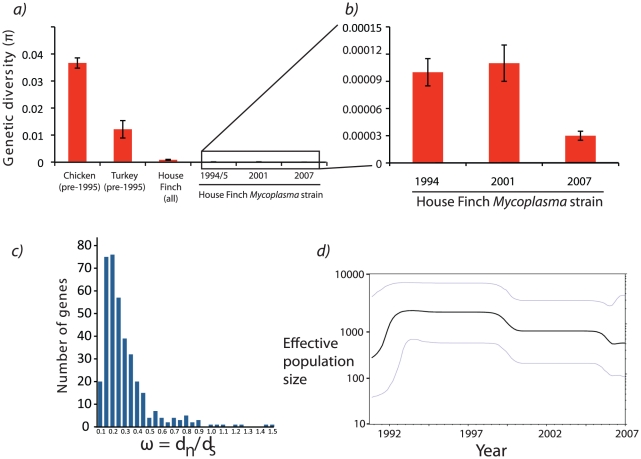
Patterns of polymorphism among *Mycoplasma gallisepticum* isolates collected from House Finches. a) Comparison of nucleotide diversity between historical chicken MG strains and serially sampled House Finch MG isolates for a 1.3 kb region [28]. b) Expansion of House Finch nucleotide diversity measured across the whole-genome alignment (approximately 738 kb when considering only the 12 House Finch isolates). c) Patterns of synonymous and nonsynonymous substitution for all MG isolates sequenced in this study as well as the reference. The values in this histogram reflect estimates of ω = *d*_n_/*d*_s_ across a tree including all House Finch isolates and the poultry R_low_ reference. For a full list of patterns of substitution for each gene, see Data S1 (Estimates of omega.xls). d) Bayesian skyline plot estimated from the alignment of 12 of house finch Mycoplasma strains. Although the upper and lower 95% confidence limits (gray lines) on the skyline plot are substantial, the overall trend (black line) is indicative of population growth approximately 17 years before 2007, or 1990, placing the spread of MG somewhat earlier than the first field observations in 1994. Note that time is reversed so that time proceeds from left (past) to right (most recent time of sampling).

The whole genome alignment contained strong signals of a founder event as a result of colonization of House Finches. The total nucleotide diversity (π) in the House Finch strains for the four-gene region was only 3.1% of the diversity in circulating poultry strains prior to the epizootic, and only 2.3% of the poultry diversity when considering the entire House Finch MG genome [28] (Figure 2 and Table S5). In agreement with the four-gene analysis, our whole genome sequencing showed that the four sequenced poultry isolates were much more genetically diverse than the 12 House Finch isolates, possessing a total of 13,175 SNPs as compared to only 412 SNPs among the House Finch isolates (Table S2). The House Finch MG diversity corresponds to π = 0.00014, or roughly 1 SNP every 1,800 bp. Consistent with purifying selection acting over the longer time period encompassing the divergence of House Finch and poultry MG strains (as opposed to acting after the host-switch among House Finch strains alone), there was a stronger bias against non-synonymous substitutions among the more diverged poultry strains than among the recently diverged House Finch MG strains (Table S6). Across the entire genome, only 147 (35%) of the SNPs among the House Finch isolates were phylogenetically informative; the majority (265 or 64%) appeared as singletons.

**Figure 3:**
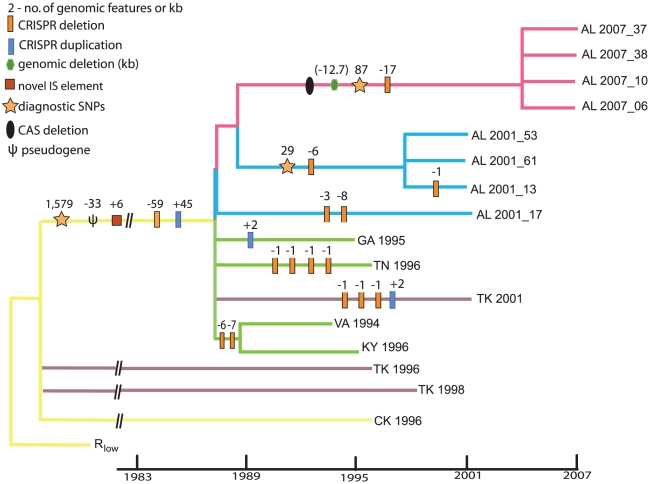
Phylogeny of *Mycoplasma gallisepticum* isolates collected at time points 1994–2007 following a host shift from poultry to House Finches. The basic topology and branch lengths of the tree come from the output for the BEAST analysis made while estimating evolutionary rates. From this tree we collapsed branches with less than 0.6 posterior probability or if there were no phylogenetically informative SNPs supporting that branch. Several strains are shown as polytomies because their genomic histories are shaped by recombination. Within the House Finch MG clade, branch lengths are proportional to time. Major genomic events are indicated on appropriate branches. The numbers of diagnostic SNPs indicated on various branches are minima. The numbers of CRISPR changes shown are only those that can be constructed with reasonable support (Figure 5); one possible reconstruction is presented.

To further quantify House Finch MG demography, we used a statistical model, the Bayesian skyline plot implemented with BEAST, that utilizes information on dates of sampling to estimate changes in genetic diversity through time [32], [33] (Text S2). The analysis is broadly consistent with field observations suggesting a mid-1990s origin followed by rapid population expansion, though it estimates that the House Finch MG lineages coalesced roughly in 1988, several years prior to the observation of sick birds in the field (estimated MRCA of the House Finch MG strains is 19.2 years prior to 2007 [95% HPD 16.9 – 21.7]; Figure 2d). Discrepancies between coalescence times and observed outbreaks in host populations have been observed for other pathogens, and could possibly be due to selective or demographic effects, or in our case low sample size [12]. Phylogenetic analysis suggests substantial turnover in the standing SNP variation between sampling intervals, with strong clustering of the 2007 strains, which are distinguished from other House Finch strains by 85 diagnostic SNPs (Figure 3). We found that one of the sequenced turkey strains, TK_2001, was highly similar in sequence to the House Finch strains and shares a number of genomic deletions and transposon insertions as well as duplications and losses of CRISPR spacers (see below) with the House Finch MG strains. This turkey strain may represent a poultry lineage close to the source lineage for House Finch MG (Figure 3).

In addition to SNPs in House Finch MG we found five large genomic deletions that occurred by 2007 and amounted to ∼42, 245 bp and encompassing 34 genes relative to the chicken R_low_ strain (Figure 1 and Figure 3, Table S7). Three of these deletions are phylogenetically informative among the 17 MG strains (Table S7), but their conflicting phylogenetic distribution underscores the presence of recombination (see next section). Two deletions totaling 9,275 bp were shared among all strains except the reference. In addition, we detected six novel IS element insertions in the House Finch MG lineage (Text S3, Table S8) and three of the genomic deletions were likely mediated by illegitimate recombination between flanking IS elements (Table S7). In addition to the 34 genes deleted as part of genomic deletions, we found evidence for pseudogenization of 19 genes relative to the chicken MG reference (Text S3, Table S9). Two genes appear to have been disrupted by transposon insertions and 17 genes were pseudogenized by frameshift or nonsense mutations (Table S9). The substantial gene losses we detected, a total of 52 genes (∼8.6%) fixed in the House Finch MG lineage, presumably as a result of the bottleneck during host switch. By contrast, we failed to find a single novel gene in House Finch MG that was not also found in the poultry MG strains (Text S5). Comparative analysis with other *Mycoplasma* genomes showed that 15% of these lost genes also lacked a homologue in the other genomes surveyed whereas 13% had a homologue in every genome (Table S9).

### Recombination and lateral gene flow

Despite the small amount of genetic variation segregating among our House Finch *Mycoplasma* samples (only 412 SNPs), it is not possible to construct a phylogenetic tree for these strains that is free of homoplasies. Although the four 2007 strains and all 2001 strains except AL_2001_17 clearly formed well defined clades based on 85 and 28 SNPs, respectively, establishing the phylogenetic relationships for the other 5 House Finch MG strains exclusively via SNPs was not possible (Text S6, Figure 3). Although a total of 16 SNPs were phylogenetically informative for the placement of these five strains, the largest cluster of SNPs that were phylogenetically consistent was seven, and overall, 13 different trees were supported by at least 3 SNPs each. Similarly, substantial homoplasy was found among the four newly sequenced poultry strains and the R_low_ reference. Although 6,152 SNPs were parsimony informative for these five strains, the unrooted tree with the best support was in conflict with 4,619 (75%) of these SNPs. These patterns are expected if sites are being shuffled by recombination or horizontal gene transfer (HGT) among isolates, and analysis of the entire data set found strong support for this (Text S4, Figures S3, S4, S5). Using the pairwise homoplasy index test [34] revealed a statistically significant signal of recombination (*p*<10^−9^). This signal comes predominantly from the four newly sequenced poultry strains because there is not enough genetic variation to make this test significant when only the House Finch strains are considered. However if we apply to the House Finch MG strains the homoplasy test by Maynard-Smith and Smith [35], which is found to perform well in situations of low nucleotide diversity [36], we again obtain a significant signal for recombination (*p*<10^−6^). We conclude that, despite a significant signal for recombination in both the poultry and House Finch strains, the House Finch MG cluster as a whole is a distinct and easily identifiable phylogenetic lineage with a long branch separating it from the poultry strains (Figure 3).

### Substitution rate and robustness to model assumptions

**Figure 4:**
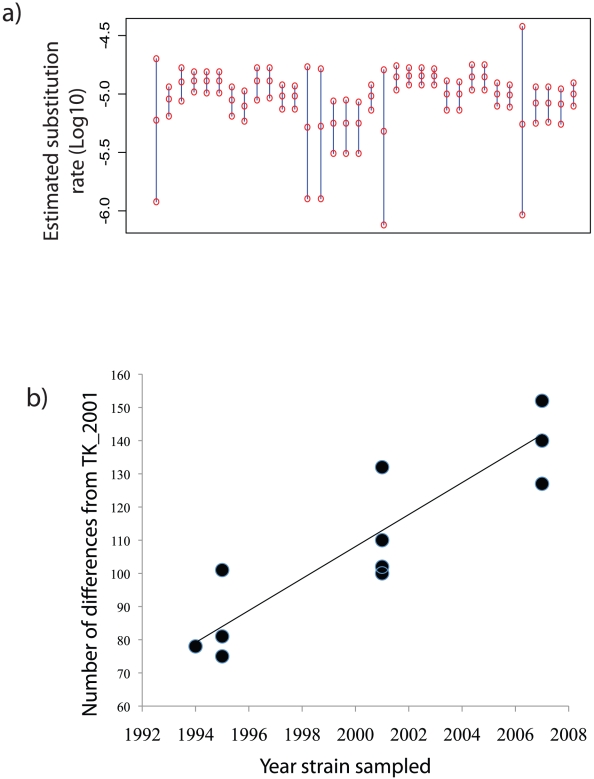
95% highest posterior density intervals on the estimated substitution rate. A) for House Finch *Mycoplasma* strains derived from 34 analyses using the different data and model combinations described in Text S2. The middle circle of each bar is the estimated mean; top and bottom circles are the upper and lower 95% bounds of each highest posterior density (HPDs). b) Root-to-tip graph of sampling date of House Finch *Mycoplasma* strains versus divergence from the closest sequence in the putative source population TK_2001. A simple regression gives an estimated substitution rate of 1.45×10^−5^, consistent with estimates from BEAST. See Text S2 and Text S7 for further information.

Coalescent analysis [32] of the 12 House Finch isolates sampled at different dates suggested an extraordinary point substitution rate of 1.02×10^−5^ substitutions per site per year (95% HPD 7.95×10−6 to 1.23× 10−5 (Text S2), consistent with earlier suggestions that *Mycoplasma* may be among the fastest evolving bacteria [37]. This rate of point substitution is not restricted to House Finch MG strains but was also found in the poultry strains when analyzed separately (Text S2), suggesting that rapid evolution was characteristic of MG prior to the House Finch epizootic. We estimated a similar substitution rate when considering only the four-gene multistrain alignment use to identify poultry strains for sequencing (Text S2). We verified that our estimate of substitution rate is robust to different protocols for SNP identification, statistical models and data sets (Figure 4; Text S7). Altogether we estimated the substitution rate within a coalescent framework on 34 combinations of SNP calling and model assumptions and found consistent estimates throughout (Text S1, Figure 4, Figure S6). In addition, we achieved a similar estimate using a Poisson regression approach as well as a root-to-tip regression (Text S7 and Figure 4).

### A possible mutator strains in House Finch MG

In addition to a high estimated substitution rate in MG, we found a mutation in the gene-encoding *UvrB* that could elevate this rate yet further. *UvrB* is an essential part of the nucleotide excision repair system, which has been posited to be the most important pathway for maintaining genomic integrity in *Mycoplasma* [38]. The mutation truncates the *UvrB* protein by three amino acids (Table S10) and raises the possibility of the origin of a mutator strain in House Finch MG [39] as the C-terminal of this protein is essential for its function [40]. Consistent with this idea, we found 14 instances of adjacent SNPs among the 12 House Finch isolates, a notable excess in an alignment with only 412 variable sites (Table S11). Moreover, 12 of these 14 are CC→TT double substitutions, which are normally repaired by the UVR system (Table S10). For 13 of the 14 doublets, both sites are inferred to have mutated on the same branch of the tree, suggesting single mutational events, and the proportion of doublet mutations involving the same base was drastically higher (92.8%) in lineages with the *UvrB* mutation as compared to those without (*p*<0.0001; Table S10). Nonetheless, these doublet mutations are not required to achieve the high rate of substitution that we measured. They account for less than 7% of the segregating variation and removal of these doublet sites does not affect the high estimated substitution rate. The *UvrB* mutation is found in all of our House Finch MG strains as well as the turkey strain TK_2001, but not in the ancestral chicken strains or the reference chicken strain. Thus, the mutation appears to have arisen on the lineage leading to the House Finch.

### Degredation and apparent functional loss of CRISPR loci in House Finch MG

**Figure 5:**
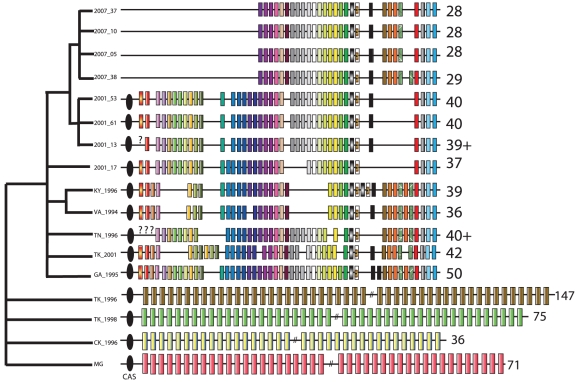
Evolution of the CRISPR locus in *Mycoplasma gallisepticum* isolates collected from House Finches, chickens, and turkeys. Numbers by each strain indicate the number of repeats in each CRISPR array. The ancestral 71-repeat CRISPR array of the chicken MG strain is shown in simplified form at bottom. Diagnostic CRISPR repeats for House Finch MG isolates are indicated in repeat-specific patterns. The black ovals signify the cluster of four CRISP-associated (CAS) genes, which are deleted in the 2007 strains. The tree at left is broadly consistent with the tree based on SNPs (Figure 3) but emphasizes strain clusters indicated by rare genomic changes and CRISPR deletions; it was constructed as described in Text S3.

In some bacterial systems, CRISPRs have a well-recognized function in bacterial immunity and defense against phage, although they may possess additional functions, such as gene regulation [41]–[44]. We extensively catalogued CRISPR repeats in the House Finch and ancestral poultry strains (Figure 5, Text S8, Table S12). In so doing we observed drastic changes in the CRISPR system between House Finch and poultry strains (Figure 5) [45]–[48]. The House Finch MG strains from 1994–96 contain up to 50 unique spacers, none of which is shared with the four divergent poultry genomes, which each contained a unique set of 36 to 147 spacer regions consistent with a high rate of turnover for a population actively acquiring new spacer sequences. We found that less than 1% of the 302 unique spacer sequences had similarity to any sequences in the House Finch MG genomes and that none of the remaining spacers had any similarity to sequences in Genbank, indicating an external source for these sequences (Text S8). Surprisingly, no novel spacer elements are present in any of the House Finch MG samples or TK_2001, indicating that the CRISPR array ceased recruiting additional spacers around the time of host switch into the House Finch. In fact, over the 13-year period of the epizootic, the number of unique spacers present in the CRISPR array of the samples decreased to 28 (Figure 5). Further evidence for degradation of the CRISPR locus following the host switch is the complete loss of the four CRISPR-associated (i.e. “CAS”) genes in all of the 2007 isolates, a loss that likely renders the CRISPR system in House Finch MG non-functional [45].

## Discussion

### Rapid substitution rate

We conducted whole-genome sequencing on a unique 13-year serial sample of *Mycoplasma* strains circulating in wild House Finches to characterize genomic changes accompanying a host shift from poultry in the mid-1990s as well as to obtain a very high substitution rate for this avian pathogen. Previous estimates using serial samples and/or the known timing of events presumably tied to the divergence of bacterial strains have generally found much lower rates. An estimate of 2.0×10^−6^ was obtained for *Staphyloccous aureus* [12], 1.1×10^−7^ for *Buchnera* [19], 7.42×10^−7^ in *Yersinia pestis* and 1.4×10^−6^ in *Heliobacter pylori* [14]. Disentangling the effects of recombination and point substitution can be challenging and some previously published substitution rates are likely to be upper bounds rather than point estimates [12]. Our estimate appears to be among the highest reported for a bacterium, and is consistent with other reports of exceptionally high substitution rates in mycoplasmas [37].

Estimates of substitution rates can be influenced by the interval over which sequences are sampled, with estimates taken from short time intervals often exceeding those taken on biogeographic or geological time scales [49]. However the small number of SNPs that we detected segregating in House Finch MG populations suggest negligible effects of multiple hits on our estimate, and our use of a coalescent model suggests that effects of ancestral polymorphism on substitution rate estimates should be adequately accounted for [32], [50]. Additionally, our estimates of substitution rate were robust to many potential complicating factors, including SNP calling protocol and whether poultry or House Finches were used as the host for sampled sequences. Given the history and genetic isolation of the House Finch MG strains, the influence of recombination or lateral gene transfer on our estimate of substitution rate is likely also minimized (Text S7).

### Rapid evolution and degradation of CRISPRs

The CRISPR dynamics we observed in House Finch MG differ from that seen in other pathogen and bacterial populations. A recent study of *Y.* pestis CRISPR arrays from 131 strains [51] indicated a slower pace of CRISPR evolution than observed in MG and pattern of evolution in which acquisition of novel sequences does not play a prominent role. This study found that in *Y. pestis* the first part of the CRISPR arrays were conserved and that over 76% of all spacer sequences derived from within the *Y. pestis* genome. Similarly, a recent study of *E. coli* and *Salmonella* genomes found that strains within 0.02% divergence typically have identical CRISPR loci [52] and that spacer sequences were often matched to elements of the *E. coli* genome. Additionally, some spacer sequences were shared between strains within a species exhibiting over 1% sequence divergence. These observations and an estimated substitution rate on the order of 10^−10^ per site per year suggested that *E. coli* strains that had diverged for 1,000 years sometimes shared identical CRISPR loci, suggesting patterns of evolution different from that expected for a rapidly changing adaptive immune system primed to combat phages, a conclusion that was supported by later work [53].

By contrast to the pattern seen in these γ-proteobacteria, none of the House Finch MG strains in this study have the same CRISPR locus despite differing at only 0.01–0.02% of sites and likely having last shared a common ancestor less than 20 years ago. Our serial sampling suggests that the loss of spacer sequences and the CRISPR system itself can take place on very short time scales in *Mycoplasma*. Unlike the patterns seen in *E. coli, Y. pestis*, and *Salmonella*, the poultry MG strains in our study did not share any spacer sequences, even though they differed by ∼1%. These strains had very large CRISPR arrays and 99% of all spacer sequences did not match any known sequence in their genome or in the databases. Therefore the MG CRISPR loci studied here differ from the those observed in some γ-proteobacteria, a group for which CRISPR dynamics can appear functionally unrelated to ecology or immunity [53]–[55].

Instead, our finding of rapid evolution and degradation of the CRISPR loci more closely resembles patterns found in other bacterial groups, particularly those in which CRISPR is involved in phage defense [56]. CRISPRs are found in only 40% of sequenced bacteria investigated thus far, and often have major roles in bacterial immunity in several lineages investigated in detail [45]. We were surprised to find a gradual degradation and ultimate apparent functional loss of the CRISPR system in House Finch MG after the host switch and a shift in CRISPR dynamics appears to be a major correlate of host switch in this system. One possible explanation for this pattern is that MG experienced release from its ancestral phage parasite community (or other mobile genetic elements such as plasmids) following introduction into the House Finch. Loss of traits upon removal of the agent of selection is a common evolutionary response, as are population expansions of animals and plants when introduced into novel habitats unaccompanied by their parasites [57].

Despite the large amount of ecological research focusing on this host-pathogen system [3]–[7], at present nothing is known about phages that infect MG or their role in its evolutionary dynamics. Therefore the hypothesis of parasite release as a driver of CRISPR loss is purely speculative. We know of no phage known to infect the Pneumoniae phylogenetic group of mycoplasmas and the few phages known to infect *Mycoplasma* have proven difficult to characterize [58]. We might expect *Mycoplasma* bacteriophages to be host-specific given that they seem to be unusual in their ability to bind to a bacterium with no cell wall and a diverse assortment of surface proteins [58]. However, we are not aware of even basic data on the degree to which *Mycoplasma* might be susceptible to the many bacteriophages that they presumably encounter in their environment. Although phage represent one possible source for these novel ∼30 bp sequences, another possible explanation for the source of the spacer sequences is that they derive from plasmids. Although unprecedented (we know of no examples of a naturally occurring plasmid in the Pneumoniae mycoplasmas), such a scenario could raise the possibility of easier genetic manipulations in MG where development of such tools has been challenging [59]. Of the many other possibilities that could explain the observed degradation of the CRISPR loci, we can at least rule out self-interference as an explanation in derived MG strains, given that there is only a single CRISPR cluster in House Finch MG [54]. Measurement of costs, possible advantages and consequences of CRISPR loss, as well as functional and evolutionary assays and surveys of phage diversity will help determine if the rapid and deadly spread of *Mycoplasma* following their expansion into the House Finch was facilitated by a lack of phage predation, a short-term advantage of CRISPR degradation or some other, possibly neutral, mechanism. Although our sequence data is suggestive, explicit functional studies will also be required to demonstrate CRISPR functionality or lack thereof in poultry and House Finch MG and its role, if any, in phage defense.

### Pseudogenization and possible mutator strains

Genome evolution of MG during its host-switch from poultry to House Finches adds to a growing list of host-switches that are successful in the complete absence of novel genes [21], [60], [61] and bacterial lineages exhibiting high rates of point substitution [14]. *Mycoplasmas* are some of the fastest evolving organisms on earth [62] having lost many of the repair mechanisms present in other bacteria [38] and this high mutation rate could help introduce deleterious mutations and contribute to the substantial level of pseudogenization that was observed in this study. The high basal substitution rate in MG may well be elevated yet further by *UvrB* mutation that we detected, a mutation that could have consequences for the long term genomic integrity of this MG lineage, particularly if it remains genetically distinct from and unable to exchange genes with the poultry MG lineages with a functional *UvrB.* Alternatively, given the short (3 amino acid) truncation of this gene in the House Finch strains, another explanation for the greatly increased number of doublet mutations in the lineage carrying the *UvrB* truncation is that selection has not had enough time to remove them as it has for poultry strains without this mutation. Although mutator strains are known to have a selective advantage in rapidly evolving laboratory and natural populations [39], [63], additional functional and experimental work will be required to determine the selective and functional effect of the mutation we have detected in *UvrB*, and over what time scales such selective effects might persist. For this and other endeavors, serial sampling of additional bacterial populations in nature will further clarify the rate at which genomes are remolded during host switches in the wild.

## Materials and Methods

### Sampling of House Finch and poultry MG strain diversity

DNA sequence data for 4 gene fragments collected from 74 strains in Ferguson et. al. [28], was combined with data from 8 strains newly sequenced in this study to yield a Large Sample Multiple Sequence Alignment (LS-MSA) 1,363 bp in length (Figure S2). We estimated nucleotide diversity and the standard deviation of this estimate within and among subgroups of these sequences using DNAsp version 4.10.9 [64] (Table S5). In estimating diversity of MG strains sampled from chickens and turkeys, we restricted analysis to those strains sampled during 1994–1996 for comparison with our earliest House Finch strains sampled in a similar time interval.

### Strain selection and genome sequencing

Twelve strains of MG isolated from House Finches in the Southeastern US were sequenced with the Roche 454 Gene Sequencer. The average coverage level was 9.4X (Table S1). Additionally, four MG strains isolated from poultry hosts and selected based on their positions in the multistrain phylogenetic tree were sequenced with the Illumina sequencing platform to an average coverage of ∼410 X (Tables S2, S3, S4, Text S1, Figure S2).

### Inference of substitutions rates, times to common ancestry, and population dynamics

Using a coalescent model and a Bayesian framework as implement in BEAST v1.52 [32] we estimated the mutation rate and times to common ancestry from a 13-taxon alignment composed of the reference MG genome and all of the House Finch MG strains whose genomes were sequenced in this study (Text S2). We also ensured that the conclusions from this inference were not sensitive to the SNP calling procedures or the choice of substitution models (Text S2, S7, Figure S6). In order to compare the mutation rate between the poultry and House Finch MG populations, these quantities were similarly estimated from the 82 taxon LS-MSA after removing nine laboratory strains from the alignment that likely experienced different population dynamics than the wild strains and had unknown sampling dates. A Poisson regression model was also used to estimate substitution rates by counting mutations along a single lineage assumed to span the dates of sampling for each strain (Text S7).

### Transposon movements, recombination, and lateral gene flow

We catalogued IS elements using BLAST and the ISFinder database [65, Text S4]. We tested for evidence of genetic recombination between MG strains using the genome sequences from our 4 poultry and 2 House Finch strains using the pairwise homoplasy index test [34] as implement in splitstree4 [66], and the homoplasy test by Maynard-Smith and Smith [35]. Further evidence for the presence of recombination and the number of nonrecombining blocks was provided by other methods (Text S6, Figures S3, S4, S5).

## Supporting Information

Figure S1 To understand the broad phylogenetic diversity of House Finch and poultry MG strains, guide our choice of poultry strains for genomic sequencing and compare mutation rates in the HF and poultry MG population, we used DNA sequence data from Ferguson et al. [28] to generate a multisequence alignment for 82 MG strains collected from four host species (Turkey, Chicken, House Finch and Gold Finch). This data, henceforth the Large Sample Multiple Sequence Alignment, LS-MSA) was composed of four gene fragments (from pvpA, mgc2, gapA and an unnamed surface lipoprotein) that when concatenated yielded approximately 1.9 kb of sequence data per strain (with the exact length of each strain varying due to small indels). We added to this dataset sequences for 8 of the 12 House Finch MG strains sequenced in this study that had complete coverage for these gene fragments. The four strains from this study not incorporated into the dataset (TN_1996, GA_1995, AL_2001_53 and AL_2007_05) were excluded because there was not enough sequencing data to accurately assemble the relevant fragments. We also excluded 3 strains from the original work[28] where we could not identify the host-animal species, leaving 82 strains in the final multiple sequence alignment. In this alignment, all the House Finch haplotypes were identical, except for the 2007 strains that differed from the others at two adjacent nucleotide positions. Certain sections of the gene fragments in the LS-MSA were polymorphic due to insertions/deletions of tandem repeats, and because there is no clear criteria by which to assign the locations of these repeats in an alignment for phylogenetic purposes, for analysis purposes we reduced the ∼1.9kb of sequence down to1,36 bp that could be confidently aligned. The tree shown is a phylogeny of 82 avian MG strains inferred from four concatenated gene-segments, totaling 1,363 bp, using Neighbor-joining in PHYLIP. Due to recombination in *Mycoplasma gallisepticum,* this single tree may not be completely representative of the organismal history of the strains from which the gene segments were sampled. However, the pattern showing poultry hosts interspersed amongst the leaves of the tree and high diversity within the MG population is also present in neighbor-joining trees separately inferred for each individual gene fragment, consistent with frequent host-shifts by MG. Strain K4366GF97_10 is from an American Goldfinch (*Carduelis tristis*), also a songbird and the chicken reference strain used to obtain the reference genome is R63_44.
(EPS)

Figure S2 Cross Validation of the 454 Sequencing Data with the Illumina Sequencing Data. Our dataset provides an opportunity to validate the SNP calls made with our 4X-19X coverage 454 data for the House Finch MG isolates by using the SNP calls made with the 294X coverage Illumina data that was generated for TK_2001. TK_2001 and the House Finch MG isolates (particularly the pre-2001 isolates) are nearly genetically identical, and SNPs for both strains were called relative to the much more distantly related strain that was used to generate the reference genome. As outlined with the unrooted tree shown in this figure. This means that most of the SNPs called for each of the House Finch isolates should also be called for the TK_2001 strain, with any unmatched SNPs likely due to either genetic divergence between the two strains or SNP calling errors. The results of this comparison are shown in Table S4. For our most stringent threshold, of the up to 6,461 SNPs that were called in our pre-2001 House Finch isolates, 99.7% of the SNPs called with the 454 data were also called with the Illumina data. This bounds the false positive rate for SNP calls in the 454 stringent data at 0.3%. However, we believe that this unmatched 0.3% is due to true genetic divergence between the strains and not sequencing errors, as these SNPs are very well supported. For example, all 21 SNPs in VA_1994 that did not match TK_2001 were supported by at least 9 reads that contained the variant, and often many more. Table S4 documents the robustness of our population genetic estimates on variations in SNP calling protocol, leading only to minor variations (∼1%) in the false positive rate for our SNP datasets. This shows that almost all of the uncertainty in estimating the mutation rate from these genomes is due to the inherent sampling variability that naturally results from the stochastic process that generated them and is not due to any variability that comes from calling SNPs in these genomes. Additionally the ratio of polymorphic to conserved sites is equivalent across all three datasets.
(EPS)

Figure S3 Illustration of the recursive method used to assign segments of the genome to phylogenetically concordant blocks. At the initialization of the algorithm the phylogenetically informative SNPs in the genome (x's in the diagram) are used to determine continuous segments that are in agreement with all possible trees. Sections of a genome in agreement with a particular tree are shown as solid colored lines over that genome segment. Note that any one SNP can be in agreement with multiple trees. If only one of two adjacent SNPs are in agreement with a tree, then half of the distance between the two SNPs is assigned to the concordant segment.
(EPS)

Figure S4 Distribution of the number of phylogenetically concordant segments in the genome and in a dataset obtained by a single random permutation of the SNPs. Block sizes are in bp.
(EPS)

Figure S5 Distribution of the size of phylogenetically concordant segments in the genome and in a dataset obtained by repeatedly creating permutations of the SNPs.
(EPS)

Figure S6 95% HPD intervals of the rate estimated in BEAST using our actual dataset, as well as 20 permutations of the data where the dates on the tips are randomly reassigned. The interval for the true dataset is shown in red, and the randomized datasets are shown in blue.
(EPS)

Table S1 Characteristics of MG isolates used in this study.
(PDF)

Table S2 SNP counts in the alignments.
(PDF)

Table S3 SNPs validated by PCR amplification and Sanger sequencing.
(PDF)

Table S4 Cross validation of 454 and Illumina data.
(PDF)

Table S5 Estimates of genetic diversity based on the LS-MSA.
(PDF)

Table S6 Patterns of synonymous and nonsynonymous substitutions.
(PDF)

Table S7 Regions of the reference genome that had been lost in House Finch MG isolates.
(PDF)

Table S8 Descriptions of six novel insertion sites of IS elements.
(PDF)

Table S9 Comparative evaluation of genes pseudogenized or deleted in the House Finch MG isolates.
(PDF)

Table S10 Mutations in the *UvrB* gene and possible effects.
(PDF)

Table S11 Instances of polymorphic adjacent SNPs among the house finch MG strains.
(PDF)

Table S12 Counts of unique and total (due to duplication) CRISPR spacers from each strain.
(PDF)

Text S1 Sequencing, alignment, and SNP calls.
(PDF)

Text S2 Inference of mutation rate, recombination, times to common ancestry, and population dynamics.
(PDF)

Text S3 Evaluating the effect of frameshift and nonsense mutations.
(PDF)

Text S4 Transposon (IS) Movements.
(PDF)

Text S5 Searching for Novel Genes in the House Finch MG isolates.
(PDF)

Text S6 Detecting recombination.
(PDF)

Text S7 Effect of recombination on the estimated substitution rate and demonstration of true temporal signal.
(PDF)

Text S8 CRISPR Analysis
(PDF)

## References

[1] Fischer J, Stallknecht D, Luttrell P, Dhondt A, Converse K (1997). Mycoplasmal conjunctivitis in wild songbirds: the spread of a new contagious disease in a mobile host population.. Emerg Infect Diseases.

[2] Nolan P, Hill G, Stoehr A (1998). Sex, size, and plumage redness predict house finch survival in an epidemic.. Proceedings of the Royal Society B-Biological Sciences.

[3] Dhondt AA, Dhondt KV, Hawley DM, Jennelle CS (2007). Experimental evidence for transmission of *Mycoplasma gallisepticum* in house finches by fomites.. Avian Pathol.

[4] Dhondt AA, Tessglia DL, Slothower RL (1998). Epidemic mycoplasmal conjunctivitis in House Finches from eastern North America.. J Wildlife Dis.

[5] Faustino C, Jennelle C, Connolly V, Davis A, Swarthout E (2004). *Mycoplasma gallisepticum* infection dynamics in a house finch population: seasonal variation in survival, encounter and transmission rate.. Ecology.

[6] Hochachka WM, Dhondt AA (2000). Density-dependent decline of host abundance resulting from a new infectious disease.. Proc Natl Acad Sci (USA).

[7] Luttrell M, Fischer J, Stallknecht D, Kleven S (1996). Field investigation of *Mycoplasma gallisepticum* infections in house finches (*Carpodacus mexicanus*) from Maryland and Georgia.. Avian Dis.

[8] Rambaut A, Pybus O, Nelson M, Viboud C, Taubenberger J (2008). The genomic and epidemiological dynamics of human influenza A virus.. Nature.

[9] Biek R, Henderson JC, Waller LA, Rupprecht CE, Real LA (2007). A high-resolution genetic signature of demographic and spatial expansion in epizootic rabies virus.. Proc Natl Acad Sci (USA).

[10] Barrick J, Yu D, Yoon S, Jeong H, Oh T (2009). Genome evolution and adaptation in a long-term experiment with *Escherichia coli*.. Nature.

[11] Harris SR, Feil EJ, Holden MT, Quail MA, Nickerson EK (2010). Evolution of MRSA during hospital transmission and intercontinental spread.. Science.

[12] Nubel U, Dordel J, Kurt K, Strommenger B, Westh H (2010). A Timescale for Evolution, Population Expansion, and Spatial Spread of an Emerging Clone of Methicillin-Resistant *Staphylococcus aureus*.. PLoS Path.

[13] Croucher NJ, Harris SR, Fraser C, Quail MA, Burton J (2011). Rapid Pneumococcal Evolution in Response to Clinical Interventions.. Science.

[14] Morelli G, Didelot X, Kusecek B, Schwarz S, Bahlawane C (2010). Microevolution of Helicobacter pylori during Prolonged Infection of Single Hosts and within Families.. PLoS Genet.

[15] He M, Sebaihia M, Lawley TD, Stabler RA, Dawson LF (2010). Evolutionary dynamics of Clostridium difficile over short and long time scales.. Proc Natl Acad Sci (USA).

[16] Holt KE, Parkhill J, Mazzoni CJ, Roumagnac P, Weill FX (2008). High-throughput sequencing provides insights into genome variation and evolution in Salmonella Typhi.. Nat Genet.

[17] Roumagnac P, Weill FX, Dolecek C, Baker S, Brisse S (2006). Evolutionary history of Salmonella Typhi.. Science.

[18] Morelli G, Song YJ, Mazzoni CJ, Eppinger M, Roumagnac P (2010). Yersinia pestis genome sequencing identifies patterns of global phylogenetic diversity.. Nat Genet.

[19] Moran N, McLaughlin H, Sorek R (2009). The dynamics and time scale of ongoing genomic erosion in symbiotic bacteria.. Science.

[20] Ochman H, Elwyn S, Moran N (1999). Calibrating bacterial evolution.. Proc Natl Acad Sci (USA).

[21] Parkhill J, Sebaihia M, Preston A, Murphy L, Thomson N (2003). Comparative analysis of the genome sequences of *Bordetella pertussis, Bordetella parapertussis* and *Bordetella bronchiseptica*.. Nat Genet.

[22] Eppinger M, Baar C, Linz B, Raddatz G, Lanz C (2006). Who ate whom? Adaptive Helicobacter genomic changes that accompanied a host jump from early humans to large felines.. PLoS Genet.

[23] Papazisi L, Gorton TS, Kutish G, Markham PF, Browning GF (2003). The complete genome sequence of the avian pathogen Mycoplasma gallisepticum strain Rlow.. Microbiol.

[24] Farmer KL, Hill GE, Roberts SR (2002). Susceptibility of a naive population of house finches to Mycoplasma gallisepticum.. J Wildlife Dis.

[25] Nolan PM, Roberts SR, Hill GE (2004). Effects of Mycoplasma gallisepticum on reproductive success in house finches.. Avian Dis.

[26] Wang Z, Baker AJ, Hill GE, Edwards SV (2003). Reconciling actual and inferred population histories in the house finch (Carpodacus mexicanus) by AFLP analysis.. Evolution.

[27] Wang Z, Farmer K, Hill GE, Edwards SV (2006). A cDNA macroarray approach to parasite-induced gene expression changes in a songbird host: genetic response of house finches to experimental infection by Mycoplasma gallisepticum.. Mol Ecol.

[28] Ferguson N, Hepp D, Sun S, Ikuta N, Levisohn S (2005). Use of molecular diversity of *Mycoplasma gallisepticum* by gene-targeted sequencing (GTS) and random amplified polymorphic DNA (RAPD) analysis for epidemiological studies.. Microbiol.

[29] Dalloul RA, Long JA, Zimin AV, Aslam L, Beal K (2010). Multi-Platform Next-Generation Sequencing of the Domestic Turkey (Meleagris gallopavo): Genome Assembly and Analysis.. PLoS Biol.

[30] Dimcheff DE, Drovetski SV, Mindell DP (2002). Phylogeny of Tetraoninae and other galliform birds using mitochondrial 12S and ND2 genes.. Mol Phyl Evol.

[31] Barker FK, Cibois A, Schikler P, Feinstein J, Cracraft J (2004). Phylogeny and diversification of the largest avian radiation.. Proc Natl Acad Sci (USA).

[32] Drummond A, Rambaut A (2007). BEAST: Bayesian evolutionary analysis by sampling trees.. BMC Evol Biol.

[33] Drummond AJ, Rambaut A, Shapiro B, Pybus OG (2005). Bayesian coalescent inference of past population dynamics from molecular sequences.. Molecular Biology and Evolution.

[34] Bruen T, Philippe H, Bryant D (2006). A simple and robust statistical test for detecting the presence of recombination.. Genetics.

[35] Maynard Smith J, Smith N (1998). Detecting recombination from gene trees.. Mol Biol Evol.

[36] Posada D, Crandall K (2001). Evaluation of methods for detecting recombination from DNA sequences: computer simulations.. Proceedings of the National Academy of Sciences of the United States of America.

[37] Woese C, Stackebrandt E, Ludwig W (1985). What are mycoplasmas: the relationship of tempo and mode in bacterial evolution.. J Mol Evol.

[38] Carvalho F, Fonseca M, Batistuzzo De Medeiros S, Scortecci K, Blaha C (2005). DNA repair in reduced genome: the mycoplasma model.. Gene.

[39] Sniegowski PD, Gerrish PJ, Lenski RE (1997). Evolution of high mutation rates in experimental populations of E. coli.. Nature (London).

[40] Moolenaar G, Franken K, Dijkstra D, Thomas-Oates J, Visse R (1995). The C-terminal region of the UvrB protein of Escherichia coli contains an important determinant for UvrC binding to the preincision complex but not the catalytic site for 3-incision.. Journal of Biological Chemistry.

[41] Levin BR (2010). Nasty Viruses, Costly Plasmids, Population Dynamics, and the Conditions for Establishing and Maintaining CRISPR-Mediated Adaptive Immunity in Bacteria.. PLoS Genet.

[42] Nozawa T, Furukawa N, Aikawa C, Watanabe T, Haobam B (2011). CRISPR Inhibition of Prophage Acquisition in Streptococcus pyogenes.. PLoS ONE.

[43] Sorek R, Kunin V, Hugenholtz P (2008). CRISPR—a widespread system that provides acquired resistance against phages in bacteria and archaea.. Nat Rev Microbiol.

[44] Vale PF, Little TJ (2010). CRISPR-mediated phage resistance and the ghost of coevolution past.. Proceedings of the Royal Society B-Biological Sciences.

[45] Sorek R, Kunin V, Hugenholtz P (2008). CRISPR—a widespread system that provides acquired resistance against phages in bacteria and archaea.. Nature Reviews Genetics.

[46] Barrangou R, Fremaux C, Deveau H, Richards M, Boyaval P (2007). CRISPR provides acquired resistance against viruses in prokaryotes.. Science.

[47] Deveau H, Barrangou R, Garneau JE, Labonte J, Fremaux C (2008). Phage response to CRISPR-encoded resistance in *Streptococcus thermophilus*.. J Bacteriol.

[48] Tyson G, Banfield J (2008). Rapidly evolving CRISPRs implicated in acquired resistance of microorganisms to viruses.. Envir Microbiol.

[49] Ho SY, Phillips MJ, Cooper A, Drummond AJ (2005). Time dependency of molecular rate estimates and systematic overestimation of recent divergence times.. Mol Biol Evol.

[50] Emerson BC (2007). Alarm bells for the molecular clock? No support for Ho et al.'s model of time-dependent molecular rate estimates.. Syst Biol.

[51] Cui Y, Li Y, Gorgé O, Platonov ME, Yan Y (2008). Insight into microevolution of Yersinia pestis by clustered regularly interspaced short palindromic repeats.. PLoS ONE.

[52] Touchon M, Rocha EPC (2010). The small, slow and specialized CRISPR and anti-CRISPR of Escherichia and Salmonella.. PLoS ONE.

[53] Touchon M, Charpentier S, Clermont O, Rocha EPC, Denamur E (2011). CRISPR Distribution within the Escherichia coli Species Is Not Suggestive of Immunity-Associated Diversifying Selection.. J Bacteriol.

[54] Diez-Villasenor C, Almendros C, Garcia-Martinez J, Mojica FJ (2010). Diversity of CRISPR loci in Escherichia coli.. Microbiol.

[55] Cady KC, White AS, Hammond JH, Abendroth MD, Karthikeyan RS (2011). Prevalence, conservation and functional analysis of Yersinia and Escherichia CRISPR regions in clinical Pseudomonas aeruginosa isolates.. Microbiol.

[56] Horvath P, Barrangou R (2010). CRISPR/Cas, the immune system of bacteria and archaea.. Science.

[57] Torchin M, Lafferty K, Dobson A, McKenzie V, Kuris A (2003). Introduced species and their missing parasites.. Nature.

[58] Waldor MK, Washington (2005). Phages: their role in bacterial pathogenesis and biotechnology; Waldor MK, Friedman DI, Adhya SL, editors.. D.C.: American Society of Microbiology Press.

[59] Lee SW, Browning G, Markham P (2008). Development of a replicable oriC plasmid for Mycoplasma gallisepticum and Mycoplasma imitans, and gene disruption through homologous recombination in M. gallisepticum.. Microbiol.

[60] Cole ST, Eiglmeier K, Parkhill J, James KD, Thomson NR (2001). Massive gene decay in the leprosy bacillus.. Nature.

[61] Parkhill J, Wren BW, Thomson NR, Titball RW, Holden MTG (2001). Genome sequence of Yersinia pestis, the causative agent of plague.. Nature.

[62] Ciccarelli F, Doerks T, Von Mering C, Creevey C, Snel B (2006). Toward automatic reconstruction of a highly resolved tree of life.. Science.

[63] Hoboth C, Hoffmann R, Eichner A, Henke C, Schmoldt S (2009). Dynamics of adaptive microevolution of hypermutable Pseudomonas aeruginosa during chronic pulmonary infection in patients with cystic fibrosis.. J Infect Disease.

[64] Rozas J, Sánchez-DelBarrio JC, Messeguer X, Rozas R (2003). DnaSP, DNA polymorphism analyses by the coalescent and other methods.. Bioinformatics.

[65] Siguier P, Perochon J, Lestrade L, Mahillon J, Chandler M (2006). ISfinder: the reference centre for bacterial insertion sequences.. Nucl Acids Res.

[66] Huson DH, Bryant D (2006). Application of phylogenetic networks in evolutionary studies.. Molecular Biology and Evolution.

[67] Hillier LDW, Marth GT, Quinlan AR, Dooling D, Fewell G (2008). Whole-genome sequencing and variant discovery in C. elegans.. Nature Methods.

[68] Brockman W, Alvarez P, Young S, Garber M, Giannoukos G (2008). Quality scores and SNP detection in sequencing-by-synthesis systems.. Genome Research.

[69] Martin D (2009). Recombination detection and analysis using RDP3.. Methods Mol Biol.

[70] Jolley K, Feil E, Chan MS, Maiden MCJ (2001). Sequence type analysis and recombinational tests (START).. Bioinformatics.

[71] Guindon S, Delsuc F, Dufayard JF, Gascuel O (2009). Estimating maximum likelihood phylogenies with PhyML.

[72] Duffy S, Holmes EC (2009). Validation of high rates of nucleotide substitution in geminiviruses: phylogenetic evidence from East African cassava mosaic viruses.. Journal of General Virology.

[73] Ley D, Berkhoff J, Levisohn S (1997). Molecular epidemiologic investigations of Mycoplasma gallisepticum conjunctivitis in songbirds by random amplified polymorphic DNA analyses.. Emerging Infectious Diseases.

[74] Ley D, Berkhoff J, McLaren J (1996). *Mycoplasma gallisepticum* isolated from house finches (*Carpodacus mexicanus*) with conjunctivitis.. Avian Dis.

[75] Tully JG, Razin S (1983). Diagnostic mycoplasmology..

[76] Farmer K, Hill G, Roberts S (2005). Susceptibility of wild songbirds to the house finch strain of Mycoplasma gallisepticum.. J Wildlife Dis.

[77] Hershberg R, Petrov DA (2010). Evidence that mutation is universally biased towards AT in bacteria.. PLoS Genet.

[78] Yang Z (2007). PAML 4: Phylogenetic Analysis by Maximum Likelihood.. Molecular Biology and Evolution.

[79] Barre A, de Daruvar A, Blanchard A (2004). MolliGen, a database dedicated to the comparative genomics of Mollicutes.. Nucleic Acids Research.

[80] Moolenaar G, Franken K, van de Putte P, Goosen N (1997). Function of the homologous regions of the Escherichia coli DNA excision repair proteins UvrB and UvrC in stabilization of the UvrBC–DNA complex and in 3 -incision.. Mutation Research-DNA Repair.

