# Correction: Ultrafast Evolution and Loss of CRISPRs Following a Host Shift in a Novel Wildlife Pathogen, *Mycoplasma gallisepticum*

**DOI:** 10.1371/annotation/b5608bc6-aa54-40a7-b246-51fa7bc4a9db

**Published:** 2012-03-05

**Authors:** Nigel F. Delaney, Susan Balenger, Camille Bonneaud, Christopher J. Marx, Geoffrey E. Hill, Naola Ferguson-Noel, Peter Tsai, Allen Rodrigo, Scott V. Edwards

Figure S3 is a incorrectly a duplicate of Figure S4. The correct Figure S3 can be viewed here: 

.

