# Supplementary material for: Ultrafast Evolution and Loss of CRISPRs Following a Host Shift in a Novel Wildlife Pathogen, Mycoplasma gallisepticum
Source: PLoS Genet. 2012 Feb 9;8(2):e1002511. doi: 10.1371/journal.pgen.1002511 (PMC3276549; doi:10.1371/journal.pgen.1002511)
Supplement: Table S1 — Characteristics of MG isolates used in this study. (PDF) [file pgen.1002511.s007.pdf]

Table S1: Isolates used

We studied 12 field isolates of *Mycoplasma* collected from House Finches in the Southeastern United States. The isolates were chosen to encompass the complete time span of the epizootic with four samples from the 1994-1996 period, four from 2001 and four from 2007. We also studied four isolates of *Mycoplasma* collected from poultry. Table S1 below shows the source of the isolates, their sequencing coverage in terms of the reference genome[1], and any alternate names the strains may have had in previous studies.

Table S1. Characteristics of MG isolates used in study

Strain Name	Host species*	Coverage	Avg. Quality Score	Date Isolated	Isolated From	Source	Alternate Name
AL_2001_13	HF	11.4	27	March 6, 2001	Lee County, Alabama	This study	
AL_2001_17	HF	8.9	18	June 27, 2001	Lee County, Alabama	This study	
AL_2001_53	HF	6.5	24	March 14, 2001	Lee County, Alabama	This study	
AL_2001_61	HF	9.5	23	February 11, 2001	Lee County, Alabama	This study	
AL_2007_05	HF	8.4	34	January 20, 2007	Lee County, Alabama	This study	
AL_2007_10	HF	4.3	37	January 20, 2007	Lee County, Alabama	This study	
AL_2007_37	HF	18.9	23	February 11, 2007	Lee County, Alabama	This study	
AL_2007_38	HF	9.8	35	February 11, 2007	Lee County, Alabama	This study	
GA_1995	HF	7.2	27	February 13, 1995	Clarke County, Georgia	[17]	K3891
KY_1996	HF	7.3	22	February 26, 1996	Kentucky	[18]	K4117
TN_1996	HF	6.8	24	January 23, 1996	Shelby County, TN	[18]	K4094
VA_1994	HF	13.9	24	June, 1994	Virginia	[19]	S11
TK_2001	Turkey	294	33.4	2001	Indiana	[5]	K5054TK01
TK_1998	Turkey	391	33.4	1998	Colorado	[5]	K4669ATK98
TK_1996	Turkey	498	33.4	1996	Missouri	[5]	K4158CTK96
CK_1996	Chicken	460	33.4	1996	Missouri	[5]	K4280CK96

*HF = House Finch

The House Finch isolates from 2001 and 2007 were obtained for this study as follows. House finches were caught in wire mesh cages placed around feeders and in mist nets. Upon capture, *Mycoplasma* samples were collected by swabbing eye conjunctiva and choanal cleft of birds displaying symptoms of disease. Swabs were immediately placed into 3 mL of SP4 media preheated to 37 C. After gentle vortexing, the swab was removed and the inoculated broth [20] was incubated at 37 C overnight. After approximately 24 hours, a 1:10 blind passage was performed for each culture which was then incubated at 37 C for 5 weeks or until a color change indicated growth [21]. Following a media color change, stocks of each isolate were made as follows: 500uL of a 1:1 solution of SP4 broth and glycerol was added to 500uL of cell culture. Samples were gently mixed and frozen at -80 C for long-term storage. DNA for sequencing was prepared by re-inoculating frozen cultures into SP4 media incubated until log phase. DNA was extracted from each sample at between passage five and seven using Qiagen DNA tissue minipreps.
