# Supplementary material for: Ultrafast Evolution and Loss of CRISPRs Following a Host Shift in a Novel Wildlife Pathogen, Mycoplasma gallisepticum
Source: PLoS Genet. 2012 Feb 9;8(2):e1002511. doi: 10.1371/journal.pgen.1002511 (PMC3276549; doi:10.1371/journal.pgen.1002511)
Supplement: Table S2 — SNP counts in the alignments. (PDF) [file pgen.1002511.s008.pdf]

Table S2. SNP counts in the final working data set comprising the 17-way alignment

	All	House Finch Strains	House Finch Strains and TK_2001	1994-1996 Strains	2001 Strains	2001 Strains excluding AL_2001_17	2007 Strains	New Poultry Strains and Reference Genome excluding TK_2001	New Poultry Strains
Total SNPs	16,398	412	469	136	152	42	37	14,400	13,175
Synonymous	9,383	122	138	37	50	12	11	8,459	7,735
Non-synonymous	5,324	246	279	85	88	24	21	4,534	4,090
Non-coding	1,729	45	53	14	15	7	5	1,441	1,377
Singletons	8,576	258	310	115	103	42	36	8,208	5,601
Phylogenetically informative within the group	7,693	152	157	20	48	0	0	6,048	7,517
Fixed SNPS (Ignoring missing data)	N/A	80	1,579	1	3	29	87	1,551	140
Fixed SNPS (Require data from all group members)	N/A	8	310	0	0	20	47	1,459	55
Fixed SNPS (Require data from all strains in study)	N/A	0	301	0	0	9	24	297	0
Fixed SNPS (Require data from all non-group members, but allows incomplete data within specified group)	N/A	2	1,485	0	0	12	36	306	0
