# Supplementary material for: Ultrafast Evolution and Loss of CRISPRs Following a Host Shift in a Novel Wildlife Pathogen, Mycoplasma gallisepticum
Source: PLoS Genet. 2012 Feb 9;8(2):e1002511. doi: 10.1371/journal.pgen.1002511 (PMC3276549; doi:10.1371/journal.pgen.1002511)
Supplement: Table S3 — SNPs validated by PCR amplification and Sanger sequencing. (PDF) [file pgen.1002511.s009.pdf]

Table S3. Sanger Sequencing Validation of SNP Calls

In the early stages of the project we validated a small subset (n = 9) of SNPs via PCR and direct sequencing of 76 sequencing reactions spread across the 12 House Finch strains. We selected these sequenced positions for two reasons. First, these sites were phylogenetically informative for the pre-2001 House Finch strains whose relationships we wished to resolve. Second, we felt these SNPs were the most suspect of all of the SNPs in our dataset as they provided conflicting phylogenetic information and so were either strong evidence for an unknown source of sequencing errors in our methods or strong evidence for recombination in this population of *Mycoplasma*. We were able to rule out sequencing error as all sequenced loci confirmed the polymorphisms identified by the 454 sequencing (71 of 76 loci matched the 454 sequencing data, and 5 of 76 provided data for strains that did not have adequate coverage at that position in the original 454 data).

Table S3. SNPs Validated by PCR amplification and Sanger Sequencing

Strain	Position	Sanger bp	Reference bp	454 bp
AL_2001_13	170360	C	C	C
AL_2001_17	170360	T	C	T
AL_2001_61	170360	C	C	C
AL_2007_05	170360	T	C	T
AL_2007_10	170360	T	C	T
AL_2007_37	170360	T	C	T
AL_2007_38	170360	T	C	T
GA_1995	170360	C	C	C
KY_1996	170360	C	C	C
TN_1996	170360	C	C	N
VA_1994	170360	C	C	C
AL_2001_13	174643	C	C	C
AL_2001_17	174643	C	C	C
AL_2001_53	174643	C	C	C
AL_2001_61	174643	C	C	C
AL_2007_05	174643	T	C	T
AL_2007_10	174643	T	C	T
AL_2007_37	174643	T	C	T
AL_2007_38	174643	T	C	T
GA_1995	174643	C	C	C
KY_1996	174643	T	C	T
TN_1996	174643	C	C	C
VA_1994	174643	C	C	C
AL_2001_13	580857	G	G	G
AL_2001_61	580857	G	G	G
AL_2007_05	580857	T	G	T
AL_2007_37	580857	T	G	T
AL_2001_13	691180	G	G	G

AL_2001_17	691180	A	G	A
AL_2001_61	691180	G	G	G
AL_2001_61	691180	G	G	G
AL_2007_05	691180	A	G	N
AL_2007_10	691180	A	G	A
AL_2007_37	691180	A	G	A
AL_2007_38	691180	A	G	A
GA_1995	691180	G	G	G
KY_1996	691180	G	G	G
TN_1996	691180	G	G	N
AL_2001_17	716811	C	C	C
AL_2007_05	716811	C	C	C
AL_2007_37	716811	C	C	C
AL_2007_38	716811	C	C	C
TN_1996	716811	C	C	C
VA_1994	716811	C	C	C
AL_2001_13	720901	T	T	T
AL_2001_17	720901	T	T	T
AL_2001_53	720901	T	T	T
AL_2001_61	720901	T	T	T
GA_1995	720901	T	T	T
TN_1996	720901	T	T	T
AL_2001_13	853947	A	G	A
AL_2001_17	853947	G	G	G
AL_2001_53	853947	A	G	A
AL_2001_61	853947	A	G	A
AL_2007_05	853947	G	G	G
AL_2007_10	853947	G	G	N
AL_2007_37	853947	G	G	G
AL_2007_38	853947	G	G	G
GA_1995	853947	G	G	G
KY_1996	853947	A	G	A
TN_1996	853947	G	G	G
VA_1994	853947	A	G	A
AL_2001_13	909457	A	C	A
AL_2001_61	909457	A	C	A
AL_2007_05	909457	C	C	C
AL_2007_37	909457	C	C	C
AL_2007_38	909457	C	C	C
VA_1994	909457	C	C	C
AL_2001_13	973203	G	G	G
AL_2001_17	973203	G	G	G
AL_2001_53	973203	G	G	N
AL_2001_61	973203	G	G	G
AL_2007_37	973203	A	G	A
AL_2007_38	973203	A	G	A
GA_1995	973203	G	G	G
VA_1994	973203	G	G	G

.
