# Supplementary material for: Ultrafast Evolution and Loss of CRISPRs Following a Host Shift in a Novel Wildlife Pathogen, Mycoplasma gallisepticum
Source: PLoS Genet. 2012 Feb 9;8(2):e1002511. doi: 10.1371/journal.pgen.1002511 (PMC3276549; doi:10.1371/journal.pgen.1002511)
Supplement: Table S4 — Cross validation of 454 and Illumina data. (PDF) [file pgen.1002511.s010.pdf]

Table S4. Cross validation of the 454 SNP calls using the Illumina SNP calls

Alignment File Name **Stringent_2010_Masked_4_Val.fna**

Strain	TN_1996	GA_1995	KY_1996	VA_1994	AL_2001_53	AL_2001_17	AL_2001_61	AL_2001_13	AL_2007_10	AL_2007_05	AL_2007_38	AL_2007_37
Differences from Reference	3129	2613	5206	6482	4044	5682	5327	5770	3772	3260	5766	6732
Differences Shared with TK_2001	3121	2604	5194	6460	4022	5621	5292	5737	3723	3212	5694	6642
% Identical SNP calls	99.7%	99.7%	99.8%	99.7%	99.5%	98.9%	99.3%	99.4%	98.7%	98.5%	98.8%	98.7%
Singletons for Strain	6	7	6	15	3	54	9	10	3	3	2	7

Alignment File Name **Stringent_Moderate_v2_2010_Masked_4_Val.fna**

Strain	TN_1996	GA_1995	KY_1996	VA_1994	AL_2001_53	AL_2001_17	AL_2001_61	AL_2001_13	AL_2007_10	AL_2007_05	AL_2007_38	AL_2007_37
Differences from Reference	5402	4763	7012	7482	6118	7269	7120	7347	5722	5379	7181	7428
Differences Shared with TK_2001	5352	4690	6972	7437	6045	7173	7053	7282	5628	5275	7067	7307
% Identical SNP calls	99.1%	98.5%	99.4%	99.4%	98.8%	98.7%	99.1%	99.1%	98.4%	98.1%	98.4%	98.4%
Singletons for Strain	26	58	15	17	29	65	9	5	9	21	7	4

Alignment File Name **Moderate_2010_Masked_4_Val.fna**

Strain	TN_1996	GA_1995	KY_1996	VA_1994	AL_2001_53	AL_2001_17	AL_2001_61	AL_2001_13	AL_2007_10	AL_2007_05	AL_2007_38	AL_2007_37
Differences from Reference	6411	5875	7336	7682	6719	7526	7487	7638	6191	6380	7439	7598
Differences Shared with TK_2001	6306	5699	7262	7615	6553	7399	7374	7545	6017	6186	7291	7456
% Identical SNP calls	98.4%	97.0%	99.0%	99.1%	97.5%	98.3%	98.5%	98.8%	97.2%	97.0%	98.0%	98.1%
Singletons for Strain	70	151	36	25	108	81	38	16	77	78	16	6
