# Supplementary material for: Ultrafast Evolution and Loss of CRISPRs Following a Host Shift in a Novel Wildlife Pathogen, Mycoplasma gallisepticum
Source: PLoS Genet. 2012 Feb 9;8(2):e1002511. doi: 10.1371/journal.pgen.1002511 (PMC3276549; doi:10.1371/journal.pgen.1002511)
Supplement: Table S5 — Estimates of genetic diversity based on the LS-MSA. (PDF) [file pgen.1002511.s011.pdf]

## Table S5. Estimates of genetic diversity (π) in subgroups of MG strains sampled from different host species* in the LS-MSA

| Host species, year | N | bp | π | Standard Deviation |
|---|---|---|---|---|
| All | 73 | ~1362 | 0.01963 | 0.00106 |
| Chicken, all | 26 | ~1362 | 0.01888 | 0.00171 |
| Chicken, 1994-1996, inclusive | 4 | ~1362 | 0.01853 | 0.00397 |
| Chicken, 1994-1996 (no Australia samples) | 2 | ~1362 | 0.02428 | 0.01214 |
| Chicken, post-1996 | 18 | ~1362 | 0.01737 | 0.00191 |
| All turkey | 31 | ~1362 | 0.02253 | 0.00193 |
| Turkey, all | 33 | ~1362 | 0.02203 | 0.00159 |
| Turkey,1994-1996, inclusive | 10 | ~1362 | 0.01634 | 0.00161 |
| Turkey, post-1996 | 21 | ~1362 | 0.02332 | 0.00201 |
| House finch, all | 14 | ~1362 | 0.00057 | 0.00019 |
| **House Finch, this study** | **12** | **743,011** | **0.00014** | **0.00001** |
| **1994-1996** | **4** | **743,011** | **0.00010** | **0.00003** |
| **2001** | **4** | **743,011** | **0.00011** | **0.00004** |
| **2007** | **4** | **743,011** | **0.00003** | **0.00001** |

Data from this study (bold) and from Ferguson et al. 2005 (5).
