# Supplementary material for: Ultrafast Evolution and Loss of CRISPRs Following a Host Shift in a Novel Wildlife Pathogen, Mycoplasma gallisepticum
Source: PLoS Genet. 2012 Feb 9;8(2):e1002511. doi: 10.1371/journal.pgen.1002511 (PMC3276549; doi:10.1371/journal.pgen.1002511)
Supplement: Table S6 — Patterns of synonymous and nonsynonymous substitutions. (PDF) [file pgen.1002511.s012.pdf]

Table S6: Patterns of synonymous and nonsynonymous substitutions

We compared the frequencies of non-synonymous, synonymous and non-protein coding SNPs in the House Finch and poultry populations by comparing three groups of SNPs. The first type were polymorphisms that likely arose in the House Finch MG lineage, as they are fixed in the poultry MG strains but are polymorphic amongst the House Finch ones. The second group are those SNPs that likely arose in the poultry MG population, as they show the opposite pattern and are fixed in the House Finch strains. We also examined SNPs that represented fixed differences between the two populations and likely arose on the lineage separating the poultry and House Finch MG populations. 35 SNPs were excluded from categorization because they were polymorphic in both the poultry and House Finch populations. Finally, we obtained expected numbers of the three types of mutations by simulating mutations in the genome using the maximum a posteriori parameters for the HKY substitution model inferred from our earlier BEAST analysis (Text S2).

Observed and expected number of SNPs in various comparisons among strains.

	Polymorphic within House Finch strains	Polymorphic within poultry strains	Fixed Differences	Simulated
Synonymous	28.5%	58.0%	27.8%	25.7%
Nonsynonymous	59.9%	31.6%	40.5%	63.8%
Non-protein Coding	11.6%	10.4%	31.6%	10.5%
Total SNPs	379	15,940	79	10,000

By converting table S6 into a contingency table, one can reject the assumption that the mutations are distributed as one would expect under neutrality as defined by the simulated distribution in the poultry population, but not in the House Finch population. ($p_{poultry}$<2.2e-16, p_{HF}=.28), which is consistent with other studies that have shown very recently diverged pathogens tend to evolve neutrally [22].

To obtain estimates of the distribution of dN/dS values for each gene within MG from all of our samples using PAML v. 4.2b[23]. For each gene, we used the maximum clade credibility tree from our BEAST analysis (Test S2) and for those genes that contained both non-synonymous and synonymous mutations we used PAML to estimate the dn/ds (omega) ratio. These data are summarized in Fig. 2c of the main text.
