# Supplementary material for: Ultrafast Evolution and Loss of CRISPRs Following a Host Shift in a Novel Wildlife Pathogen, Mycoplasma gallisepticum
Source: PLoS Genet. 2012 Feb 9;8(2):e1002511. doi: 10.1371/journal.pgen.1002511 (PMC3276549; doi:10.1371/journal.pgen.1002511)
Supplement: Table S7 — Regions of the reference genome that had been lost in House Finch MG isolates. (PDF) [file pgen.1002511.s013.pdf]

## Table S7. Regions of the reference genome that had been lost in House Finch MG isolates

We searched for genes in the reference genome that were not present in the House Finch MG isolates. The 454 contigs assembled from our pooled 2007 samples were mapped to the reference genome using Megablast and any portion that aligned with greater than 95% similarity and over 100 bp in length to a section of the reference genome was considered to represent that section. We then searched for any section of the reference genome longer than 200 bp in length that was not represented by some of the reads in our sample. Unrepresented segments were then further investigated to confirm the deletion, determine the likely mechanism by which it occurred and the starting and ending points in the coordinates given by the reference genome. For the reasons given previously, any putative deletions that appeared in the VlhA regions were not investigated further in this study, even though these regions likely experienced deletions relative to the reference MG strain.

The list of reconstructed deletions in House Finch MG isolates from this analysis is shown in the Table S7; in total they account for ~42 kb of the reference genome being lost and are responsible for the deletion of a total of 34 genes. Three of these deletions are hypothesized to have occurred via recombination between IS elements. Two of the large deletions (numbers 3 and 5) could clearly be identified because no reads representing the deleted sequence were present and because a contig could be formed that spanned the deletion. However, three of the deletions (numbers 1, 3 and 5) were clearly mediated by an IS element insertion followed by a non-homologous recombination-mediated deletion. As these events are caused by recombination between non-homologous sequences, the exact location of the recombination point is unknown and only approximate coordinates are given in the Table S7. All of the deletions found were present in all of our other HF strains, except for the 12.7 kb deletion which was unique to the 2007 isolates.

| Deletion number | Approx. start | Approx. end | Deletion size (bp) | Deletion mediated by recombination between IS elements? | Distribution |
|---|---|---|---|---|---|
| 1 | 124,815 | 126,674 | 1,859 | Yes | All strains except $R_{low}$ |
| 2 | 137,173 | 138,833 | 1,660 | No | All strains except $R_{low}$ and CK_1996 |
| 3 | 369,420 | 388,013 | 18,593 | Yes | All strains except $R_{low}$ and TK_1996 |
| 4 | 912,433 | 925,150 | 12,717 | No | Only in 2007 House Finch strains |
| 5 | 938,560 | 945,976 | 7,416 | Yes | All strains except $R_{low}$ |
| Total deleted: ~42,245 bp | | | | | |
