# Supplementary material for: Ultrafast Evolution and Loss of CRISPRs Following a Host Shift in a Novel Wildlife Pathogen, Mycoplasma gallisepticum
Source: PLoS Genet. 2012 Feb 9;8(2):e1002511. doi: 10.1371/journal.pgen.1002511 (PMC3276549; doi:10.1371/journal.pgen.1002511)
Supplement: Table S8 — Descriptions of six novel insertion sites of IS elements. (PDF) [file pgen.1002511.s014.pdf]

Table S8. Descriptions of six novel insertion sites of IS elements and insert characteristics for House Finch MG strains.

	Approximate Location	Sides Present	Target Gene	Description of Insertion Area
A	124818	5’	MGA_0801	Potential C-terminal fragment of subtilisin like protease
B	295023	Both	None	This section of the genome is unannotated. The location is 1,047 and 276 bp away from the genes on either side.
C	464795	Both	MGA_1220	ArcA, a predicted arginine deiminase
D	537089	Both	None	This landed inside a pseudo-gene that formerly was an acetyl-CoA hydrolase/transferase
E	560163	Both	None	This is 201 bp and 167 bp away from the nearest genes on either side.
F	938560	5’	None	This is 142 bp and 151 bp away from the genes on either side of this insertion.
