# Supplementary material for: Ultrafast Evolution and Loss of CRISPRs Following a Host Shift in a Novel Wildlife Pathogen, Mycoplasma gallisepticum
Source: PLoS Genet. 2012 Feb 9;8(2):e1002511. doi: 10.1371/journal.pgen.1002511 (PMC3276549; doi:10.1371/journal.pgen.1002511)
Supplement: Table S9 — Comparative evaluation of genes pseudogenized or deleted in the House Finch MG isolates. (PDF) [file pgen.1002511.s015.pdf]

Table S9. Genes pseudogenized or deleted in the House Finch MG isolates and their status in other *Mycoplasma* genomes.

Among the 12 House Finch isolates we identified 34 genes that had been removed by a deletion, 2 that had been disrupted by a transposon insertion (including one that was deleted following this insertion) and 17 genes that had been pseudogenized by frameshift or nonsense mutations, for a total of 52 genes . We sought to evaluate if these genes were unique to the reference MG genome by evaluating if they had any homologues in any of the 20 Mollicute genomes available has determined by the Molligen Database [24]. We found that 5 of the 33 genes (15%) lost by a deletion lacked a homologue in at least one other genome, while 3 of the 17 genes lost by pseudogenization in the House Finch isolates (~18%) lacked a homologue in the other genomes. We also checked whether any of the genes that were lost in the House Finch isolates had homologues in every one of the 13 *Mycoplasma* genomes available in the database, and thus could be considered "core" genes. We found that of the 229 genes in the reference genome that had a homologue in all of the other genomes, 7 of these had been lost by a combination of 1 deletion and 3 frameshift mutations in the House Finch MG strains.

Table S9. Genes pseudogenized or deleted in the House Finch MG isolates and their status in other *Mycoplasma* genomes.

Gene ID	Start	End	How Lost	No Homology to other *Mycoplasma* genomes	Homology to all other *Mycoplasma* genomes	Gene Name	Product
MGA_0625a	5159	6077	Disruptor Mutation	FALSE	FALSE		ABC-type multidrug/protein/lipid (MdlB-like) transport system component domain protein
MGA_0626	6392	8294	Disruptor Mutation	FALSE	TRUE		ABC-type multidrug/protein/lipid (MdlB-like) transport system component
MGA_0641	14480	15212	Disruptor Mutation	FALSE	FALSE	glpF	glycerol uptake facilitator protein GlpF
MGA_0648	18209	20462	Disruptor Mutation	FALSE	FALSE		conserved lipoprotein
MGA_0656	30264	30948	Disruptor Mutation	TRUE	FALSE		unique hypothetical lipoprotein
MGA_0686	50592	52587	Disruptor Mutation	FALSE	TRUE	uvrB	excinuclease ABC subunit B
MGA_0687	52631	53789	Disruptor Mutation	FALSE	FALSE	pstS	ABC-type phosphate transport system periplasmic phosphate binding protein
MGA_0801	124459	125605	Deletion/IS Insertion	FALSE	FALSE		Subtilisin-like serine protease domain protein
MGA_0802	125682	126432	Deletion	TRUE	FALSE		Subtilisin-like serine protease domain protein
MGA_0815	137104	139078	Deletion	FALSE	FALSE		Subtilisin-like serine protease
MGA_1037	332335	334084	Disruptor Mutation	FALSE	FALSE		conserved hypothetical membrane protein
MGA_1328	369554	369794	Deletion	FALSE	TRUE	deoC_1	Deoxyribose-phosphate aldolase domain protein
MGA_1081	369839	371102	Deletion	FALSE	FALSE		putative transposase

MGA_1083	371070	371919	Deletion	FALSE	FALSE		HAD superfamily hydrolase Cof
MGA_1085	371929	373567	Deletion	FALSE	FALSE		conserved hypothetical protein
MGA_1087	373576	374212	Deletion	FALSE	FALSE		conserved hypothetical protein
MGA_1088	374241	374925	Deletion	FALSE	TRUE		ABC transporter ATPase component
MGA_1089	374908	376459	Deletion	FALSE	FALSE		ABC transporter permease domain protein
MGA_1091	376555	376891	Deletion	FALSE	FALSE		putative signal peptidase I
MGA_1092	376969	377530	Deletion	FALSE	TRUE		Elongation factor G domain protein
MGA_1100	379435	380476	Deletion	FALSE	TRUE	asnS_2	Asparaginyl-tRNA synthetase
MGA_1102	380479	382111	Deletion	FALSE	FALSE		conserved hypothetical membrane protein
MGA_1103	382094	384026	Deletion	FALSE	FALSE		predicted integral membrane methylase-domain protein
MGA_1347	384502	384670	Deletion	FALSE	FALSE		putative transposase domain protein
MGA_1106	384754	384946	Deletion	TRUE	FALSE		putative transposase domain protein
MGA_1107	384995	386495	Deletion	FALSE	FALSE		conserved hypothetical RmuC-domain protein
MGA_1108	386618	387119	Deletion	FALSE	FALSE		putative transposase domain protein
MGA_1109	387260	388307	Deletion	FALSE	FALSE		putative transposase domain protein
MGA_1220	464277	465489	IS Insertion	FALSE	FALSE	arcA_1	Arginine deiminase
MGA_1263	507145	507823	Disruptor Mutation	FALSE	FALSE	beta-pgm	putative beta-phosphoglucomutase (beta-PGM)
MGA_1283	520043	520808	Disruptor Mutation	FALSE	FALSE		PTS system mannitol-specific (MtlA)-like IIB domain protein
MGA_1305	536425	536824	Disruptor Mutation	FALSE	FALSE	maoC	MaoC-like dehydratase
MGA_0135	652030	653536	Disruptor Mutation	FALSE	TRUE	potA	ABC-type spermidine/putrescine import ATP-binding protein potA
MGA_0137	653891	655376	Disruptor Mutation	TRUE	FALSE		unique hypothetical protein
MGA_1361	747656	747986	Disruptor Mutation	FALSE	FALSE		unique hypothetical protein
MGA_1354	876961	877114	Disruptor Mutation	FALSE	FALSE		hypothetical protein
MGA_0508	910763	912797	Deletion	FALSE	FALSE	fruA	PTS system fructose-specific enzyme EIIABC component

MGA_0512	912799	913246	Deletion	TRUE	FALSE		hypothetical protein
MGA_0514	913193	914144	Deletion	FALSE	FALSE	manA	mannose-6-phosphate isomerase (phosphomannose isomerase)
MGA_0516	915226	916669	Deletion	TRUE	FALSE		unique hypothetical protein
MGA_0517	916577	917954	Deletion	FALSE	FALSE		Subtilisin-like serine protease domain protein
MGA_0518	917874	918705	Deletion	TRUE	FALSE		Subtilisin-like serine protease domain protein
MGA_0519	919247	923060	Deletion	FALSE	FALSE		Csn1 family CRISPR-associated protein
MGA_0523	923127	924054	Deletion	FALSE	FALSE	cas1	CRISPR-associated protein Cas1
MGA_0525	924040	924370	Deletion	FALSE	FALSE	cas2	CRISPR-associated protein Cas2
MGA_0526	924369	925134	Deletion	FALSE	FALSE		conserved hypothetical protein
MGA_0537	938710	941338	Deletion	FALSE	FALSE	hsdM	type I restriction-modification system methyltransferase (M) subunit
MGA_0539	941547	942165	Deletion	FALSE	FALSE	hsdS_1	type I restriction-modification system specificity (S) subunit domain protein
MGA_0540	942139	942724	Deletion	FALSE	FALSE	hsdS_2	type I restriction-modification system specificity (S) subunit domain protein
MGA_0541	942734	945890	Deletion	FALSE	FALSE	hsdR	type I site-specific restriction-modification system restriction (R) subunit (deoxyribonuclease)
MGA_0567	970243	970549	Disruptor Mutation	TRUE	FALSE		unique hypothetical protein
MGA_0586	987980	990098	Disruptor Mutation	FALSE	FALSE		conserved hypothetical protein
