# Supplementary material for: Ultrafast Evolution and Loss of CRISPRs Following a Host Shift in a Novel Wildlife Pathogen, Mycoplasma gallisepticum
Source: PLoS Genet. 2012 Feb 9;8(2):e1002511. doi: 10.1371/journal.pgen.1002511 (PMC3276549; doi:10.1371/journal.pgen.1002511)
Supplement: Table S10 — Mutations in the UvrB gene and possible effects. (PDF) [file pgen.1002511.s016.pdf]

# Table S10: Mutations in the *UvrB* Gene and Possible Effects

The *UvrB* gene in every house finch MG strain sampled contains a mutation that truncates the final 3 amino acids of the protein, and this mutation is also present in the closely related TK_2001. The DNA encoding the C-terminal of this amino acid contains a 2 time repeat of the sequence “TAAG” and this mutation introduced one additional repeat of this sequence as a 4 bp insertion. The effect of this 4 bp insertion was to introduce an early “TAA” stop codon and thereby truncate the protein by 3 amino acids as shown below.

Comparison of the C-Terminals in the *UvrB* gene

```
House Finch MG Isolates  …KMIEDLRNEMLEAAKNQNYEHAASLRDLIIELETQQLSK*
Reference MG Genome      …KMIEDLRNEMLEAAKNQNYEHAASLRDLIIELETQQLSKTNK*
```

*UvrB* is an integral part of the cell’s DNA excision repair system and functions by forming associations with *UvrA* and *UvrC* during the repair process. Experimental work with the *UvrB* protein from E. coli has shown that the C-terminal of this protein is essential for the protein to associate with *UvrC* and allow a repair to occur [25,26]. However, the house finch MG protein has lost only the final 3 amino acids, and so the specific effect of this mutation cannot be determined from past functional or comparative work.

DNA excision repair is responsible for the repair of pyrimidine dimers, and one signature that these types of mutations have not been repaired along an evolving lineage is the presence of “CC” to “TT” mutations (or “GG” to “AA” if the effect of the mutation is viewed from the other strand). To investigate if the rate of these mutations is elevated in the house finch MG samples, we compared the characteristics of adjacent SNPs that are found segregating amongst the house finch and TK_2001 MG samples to those adjacent SNPs that are polymorphic amongst the reference genome and the other poultry strains. This comparison is shown in table S14.

This comparison showed many features that suggested inhibition of the nucleotide excision repair system within the house MG. The majority of the double mutations within the house finch MG could be identified as involving a “CC” to ”TT” substitution on one of the strands of DNA. Among the house finch MG samples, 14 pairs of SNPs were adjacent to each other (Table S11). Of these, 13 could be parsimoniously identified as having occurred on a single, and the same, branch of the tree, and 12 of these could be defined (using the reference and poultry strains to identify the derived allele) as a “CC” to ”TT” substitution. Of the two remaining adjacent SNP pairs (at reference positions 667,905 and 715,595), one involved two mutations that occurred on separate branches on the tree, such that no genotype contained a copy of both derived alleles, and another involved an “AA” to “TT” transition. Also suggestive of an increase in the mutation rate for paired bases is the high number of adjacent SNPs given the small number of total SNPs within the HF samples. The percentage of SNPs that are adjacent to each other is expected to increase with the total number of SNPs in an alignment. However, despite having a much smaller number of SNPs, those that were polymorphic among the house finch MG strains contained a greater proportion of adjacent SNPs (Table S11).

We tested for an increase in the number of paired substitutions that involved a substitution from two identical bases to two identical bases of a different type. A contingency table for this analysis was constructed by counting only the adjacent SNPs that appeared in pairs (excluding SNPs that appeared in adjacent groups of three or more, as well as SNPs with over 2 types segregating). The frequency of identical conversions in each group was then compared and found to be significantly different ($p < 0.00001$). This analysis is slightly complicated because at one of the positions containing adjacent SNPs in the house finch MG samples, position 667,905 in the reference genome coordinates, the ancestral sequence "CC" sequence has mutated in one strain to create a "CC"-> "CA" substitution, while on the branch leading to the 2007 strains it has mutated to create a "CC"-> "TT" substitution. Although this made the classification of this pair ambiguous, the frequency difference for these types of mutations suggests that either classification still results in a significant difference, though for clarity we presented it as an identical pair substitution in table S14.

## Table S10 – Comparison of adjacent SNPs within the house finch MG to those between the house finch MG and the reference genome.

| | **SNPs polymorphic amongst strains without the *UvrB* mutation but fixed amongst strains that have it** | **SNPs that are polymorphic amongst the strains with the *UvrB* mutation.** |
|---|---|---|
| Total SNPs | 16,959 | 420* |
| SNPs adjacent to another SNP (percentage of total SNPs) | 1,458 (8.5%) | 28 (6.8%) |
| Adjacent Pairs of SNPs (excluding >3 SNPs in a row) | 641 | 14 |
| Adjacent pairs with a conversion of an identical pair to an identical pair (e.g. "CC"->"TT"); (percentage of total adjacent pairs) | 42 (6.6%) | 13 (92.8%) |
| Adjacent pairs with non-identical conversions (eg. "AA"->"TC", "AT"->"GC" or "GC" ->"CC") (percentage of total adjacent pairs) | 599 (93.4%) | 1 (7.2%) |
