# Supplementary material for: Ultrafast Evolution and Loss of CRISPRs Following a Host Shift in a Novel Wildlife Pathogen, Mycoplasma gallisepticum
Source: PLoS Genet. 2012 Feb 9;8(2):e1002511. doi: 10.1371/journal.pgen.1002511 (PMC3276549; doi:10.1371/journal.pgen.1002511)
Supplement: Table S11 — Instances of polymorphic adjacent SNPs among the house finch MG strains. (PDF) [file pgen.1002511.s017.pdf]

Table S11. Instances of polymorphic adjacent SNPs among the house finch MG strains.

	Position of double SNP in Reference Coordinates													
**Strain**	14,966	61,514	76,728	120,043	169,641	225,915	241,224	303,492	315,466	572,038	667,905	688,985	715,595	803,438
R Low	GG	CC	GG	GG	CC	CC	AA	GG	CC	GG	CC	CC	TC	GG
TN_1996		CC	GG	GG	CC	CC	AA	GG	CC	GG	CC	CC	TC	GG
VA_1994	GG	CC	GG	GG	CC	CC	AA	GG	CC	GG	CC	CC	TC	AA
KY_1996	GG	CC	GG	GG	CC	CC	AA	GG	CC	GG	CC	CC	TC	AA
GA_1995	GG	CC	GG	GG	CC	CC	AA	GG	TT	GG	CC	CC	TC	AA
AL_2001_53	GG	CC	GG	GG	CC	CC	AA	GG	CC	GG	CA	CC	TT	AA
AL_2001_17	AA	CC	AA	GG	CC	CC	AA	AA	CC	AA	TT	TT	CC	AA
AL_2001_61	GG	CC	GG	AA	CC	CC	AA	GG	CC	GG	CA	CC	TT	AA
AL_2001_13	GG	CC	GG	GG	CC	CC	AA	GG	CC	GG	CA	CC	TT	AA
AL_2007_10	GG	TT	GG	GG	TT	TT	TT	GG	CC	GG	CA	CC	TT	AA
AL_2007_05	GG	TT	GG	GG	TT	TT	TN	GG	CC	GG	CA	CC	TT	AA
AL_2007_38	GG	TT	GG	GG	TT	TT	TN	GG	CC	GG	CA	CC	TT	AA
AL_2007_37	GG	TT	GG	GG	TT	TT	TN	GG	CC	GG	CA	CC	TT	AA
TK_2001	GG	CC	GG	GG	CC	CC	AA	GG	CC	GG	CC	CC	TT	AA
CK_1996	GG	CC	GG	GG	CC	CC	AA	GG	CC	GG	CC	CC	TC	GG
TK_1998	GG	CC	GG	GG	CC	CC	AA	GG	CC	GG	CC	CC	TC	GG
TK_1996	GG	CC	GG	GG	CC	CC	AA	GG	CC	GG	CC	CC	TC	GG
