# Supplementary material for: Ultrafast Evolution and Loss of CRISPRs Following a Host Shift in a Novel Wildlife Pathogen, Mycoplasma gallisepticum
Source: PLoS Genet. 2012 Feb 9;8(2):e1002511. doi: 10.1371/journal.pgen.1002511 (PMC3276549; doi:10.1371/journal.pgen.1002511)
Supplement: Table S12 — Counts of unique and total (due to duplication) CRISPR spacers from each strain. (PDF) [file pgen.1002511.s018.pdf]

Table S12. Counts of unique and total (due to duplication) CRISPR spacers from each strain.

Strain	Total Unique Spacers	Total Spacers
CK_1996	66	75
TK_1998	35	36
TK_1996	93	147
Reference Genome	61	71
TK_2001	38	42
VA_1994	34	36
TN_1995	38	40
KY_1995	35	39
GA_1995	47	50
AL_2001_17	37	37
AL_2001_53	40	40
AL_2001_61	40	40
AL_2001_13	39	39
AL_2007_10	28	28
AL_2007_05	28	28
AL_2007_38	28	29
AL_2007_37	28	28
**All Strains**	**302**	**805**
