# Supplementary material for: Ultrafast Evolution and Loss of CRISPRs Following a Host Shift in a Novel Wildlife Pathogen, Mycoplasma gallisepticum
Source: PLoS Genet. 2012 Feb 9;8(2):e1002511. doi: 10.1371/journal.pgen.1002511 (PMC3276549; doi:10.1371/journal.pgen.1002511)
Supplement: Text S1 — Sequencing, alignment, and SNP calls. (PDF) [file pgen.1002511.s019.pdf]

Text S1: Sequencing, Alignment and SNP Calls

We used a mixed 454 and Illumina sequencing strategy. Genomic DNA from each House Finch strain was first nebulized and blunt-end ligated with Roche adaptors. Emulsion PCR was performed using the bulk library and these products were pyrosequenced on Roche 454 Gene Sequencer using FLX chemistry, using physical separation of isolates. Genomic DNA from the four newly sequenced poulty strains (Table S1) was sequenced by the Illumina method at the University of Utah Huntsman Cancer Institute.

Sequence data generated from the Illumina sequencing platform was used to make a multiple sequence alignment according to the following protocol. Raw sequences were first trimmed to retain only high quality sequence data. Trimmed reads over 25 bp in length were then aligned to the reference MG genome (AE015450.2), ignoring any ambiguously mapped reads using the CLC Genomics Workbench version 3.7.1 . Finally, bases were called from the consensus sequence in this alignment for each unmasked (see below) regions of the genome. Any basepair in these regions that differed from the consensus region was only called as a SNP if there were at least 4 reads at the position, if the base passed NQS (30/25) standards, and if at least 95% of all reads aligning at that position had the SNP base. To avoid errors due to runs of single bases, we also required that there be no more than 2 gaps or mismatches within an 11 bp window around the putative SNP in any read that was counted towards calling the SNP. This final criterion is effective at removing erroneous SNPs that are simply artifacts of poor sequence alignment. However, due to the high number of SNPs present in all four of our sequenced strains (10,007-10,729 differences in an alignment of ~756 kb), we would expect this to also exclude some legitimate SNPs that fell in windows with 2 or more neighboring SNPs simply by chance. For this reason, we generated a second dataset that varied this criterion by allowing up to 4 gaps or mismatches with in an 11 bp window around any position that differed from the reference genome. This dataset contained only 1% more SNPs than the original dataset, and we found that the conclusions in this paper were qualitatively unaffected by using this alternate dataset.

The sequencing data from the House Finch MG isolates generated on the Roche 454 platform data was aligned to the reference *Mycoplasma gallisepticum* genome [1] using the Mosaik aligner [2]. From this alignment a basepair for a strain was reported for each position in the reference genome if the following conditions were met. The majority base at that position had to have a center QC score of 30 or higher, and the 5 flanking bases on either side of it to each have a score of 25 or higher. We also required at least two reads before a base was called. A whole genome alignment was then generated by aligning the basepairs present from each sample at the same position in the reference genome. This whole genome alignment was then divided into sections and the alignments were manually curated by the authors. If during this process a region of the genome was found to clearly violate the Markov models we assumed for nucleotide evolution (e.g. a transposon insertion) or if the aligner was grossly in error (e.g. a section with a deletion or a slightly varying simple sequence repeat that confused the aligner) we either manually edited the relevant section or if it was not obvious what the correct alignment should be we excluded it from later analysis. We additionally validated our alignments by ensuring that the base we reported at every position in our alignments matched the base independently reported by a separate alignment algorithm. The other aligner we used was the run454Mapper program (part of the Genome Sequence FLX Data Analysis Software package available from Roche).

For both the Illumina and 454 sequencing data, we aggressively excluded repetitive segments of the genome that we believed to be inappropriate for SNP calls. In particular, the *Mycoplasma gallisepticum* genome contains a number of proteins that have a high degree of similarity with other proteins, such as the VlhA family of lipoproteins that constitute 10.4% of the reference genome, and also the Apr-E like proteins, transposases, CRISPRs, etc. This repetitive DNA is likely to undergo recombination and in some cases it is not possible to correctly align or assemble. To avoid artifacts introduced by these regions, we excluded any region of the genome over 100 bp in size that had over 85% similarity to another location in the reference genome as determined by megablast . In total, we excluded 228,875 bases (~23% of the reference, henceforth the masked segments) from our multi-isolate alignment and from our SNP calling protocol.

Alternate SNP Calling Protocols

To check the sensitivity of our results to our SNP calling method for the House Finch MG isolate data, we generated three alternate genomic alignments using different protocols and quality threshold levels. These datasets were all generated for only the unmasked portions of our genome, and are described below. All analyzes described in this paper were also performed with these alternate datasets and equivalent results were obtained.

a) Stringent Threshold - Broad Institute 454Swap SNP Calling Software

This dataset calls SNPs using software developed at the Broad Institute for 454 data [3]. The pipeline that uses this SNP calling software is completely independent of the other base calling methods we used. The quality threshold requirements for this program include:

a) A basepair needs to have reads that align to it coming from both the right and the left side.
b) A basepair must be represented by at least two reads that pass NQS thresholds.
c) No more than 33% of the reads at a position can disagree with the consensus base.

b) Moderately Stringent Threshold – Our working data set

This is our working dataset and was created as described in the preceding section. Using the protocol in which 2 gaps or mismatches within an 11 bp window were removed produced an alignment that was 756,552 bp in length. In addition, we also produced an alignment allowing up to 4 gaps or mismatches within an 11 bp window (with the level of diversity present in our poultry sample this many mutations could be expected to occasionally occur and so this may not always indicate sequencing errors). This alternate alignment of 756,574 bp contained only 1% more SNPs than the original method and use of this alignment did not affect any of the conclusions in the paper. This protocol also yielded an alignment of 738,209 bp when considering only the House Finch MG strains.

c) Moderate threshold -A variant of the moderately stringent data set

This dataset was produced exactly as above except there was no requirement that the data generated matched the data generated by the Roche 454 aligner.

Further SNP Dataset Validation

In addition to the checks described above, we also performed 76 traditional Sanger sequencing reactions of SNPs called in the House Finch MG dataset (Table S3) and confirmed that the SNPs were called correctly. Additionally, as our dataset is a composite of 454 and very high coverage Illumina sequencing data, we cross-validated our results by comparing these two datasets to each other and found excellent agreement between them (Figure S2, Table S4).
