# Supplementary material for: Ultrafast Evolution and Loss of CRISPRs Following a Host Shift in a Novel Wildlife Pathogen, Mycoplasma gallisepticum
Source: PLoS Genet. 2012 Feb 9;8(2):e1002511. doi: 10.1371/journal.pgen.1002511 (PMC3276549; doi:10.1371/journal.pgen.1002511)
Supplement: Text S2 — Inference of mutation rate, recombination, times to common ancestry, and population dynamics. (PDF) [file pgen.1002511.s020.pdf]

Text S2: Inference of mutation rate, recombination, times to common ancestry and population dynamics

Using BEAST v1.52 [4], estimates of times of common ancestors were obtained for both the 13-taxon alignment of 738 kb containing our 12 House Finch Strains and the reference genome (large alignment) as well as for 73-taxon LS-MSA of 1.3 kb, which included MG sequence data obtained from strains sampled between 1955 and 2000 [5]. To aid in the selection of the inference model and to ensure that the results based on the large alignment were qualitatively insensitive to inference model choice, we compared the estimates of the mutation rate obtained from a variety of different possible analyses. Since a population expansion was observed to occur over the sampling period, in all inference models considered we assumed a changing population size using the exponential skyline model [6], and also always assumed some form of the HKY nucleotide substitution model. Given these model choices, we also tested the effect of four additional choices, or factors, on our inference. One of these factors was the modeling choice for site heterogeneity, which we tried at three levels (HKY, HKY+Γ or HKY+Γ+I). We also varied the data by including and excluding the reference genome because it was sampled at a much earlier time point then the other strains and thus could exert a high amount of leverage on the rate estimate. Another factor was the multiple sequence alignment used, and we tested all three of our SNP calling datasets (Stringent, Moderate and Moderately Stringent). Finally, since the amount of sequencing data present for each of our strains varied, we tested whether the strains with greater coverage were biasing the results by running the analysis while allowing BEAST to average over partially observed sites, or by only analyzing sites with data for all strains.

In total this resulted in 36 ($3 \cdot 2 \cdot 3 \cdot 2 = 36$) different methods to infer the rate of evolution, and inference about the posterior distribution of the rate parameter was obtained for each of these methods from 10,000,000 MCMC samples. From this analysis, 2 of the 36 MCMC runs were unable to converge. These runs were performed with settings that essentially deprived the inference method of enough data to jointly infer the parameters in the model (e.g. the stringent dataset and the requirement that all strains have data present), and as a result the estimates were wildly varying and inaccurate (e.g. Median clock rates of 1.53e307) and the MCMC chains clearly failed to converge. A plot of the rate estimates from the 34 runs that could produce sensible results is shown in Fig. S4.

We concluded from this analysis that the rate estimate was robust to these model choices. However, the results reported in this paper are based on the model we believed to be the best, which included the reference strain to allow inference about its divergence time from the HF ancestor and, used the HKY+I model of substitution (when inferred, the posterior distribution of the Gamma parameter was identical to the prior because the low amount of diversity in the house finch MG meant there were not enough multiple mutations to estimate this parameter), and used our Moderately-Stringent dataset. For this model, we ran 8 additional chains starting from different initial trees and parameter settings, and checked that all converged to the same distribution. The results for this analysis gave an estimated mean clock rate of 1.02e-5 per year (95% HPD 7.95e-6 to 1.23e-5), an estimated date for the MRCA of the HF strains as having lived 19.2 (95% HPD 16.9 to 21.7) years prior to 2007 and estimates the common ancestor of the HF strains and the chicken reference to have occurred 599.2 (95% HPD 477.5 to 737.0) years

prior to 2007. We also used this analysis to estimate a skyline plot for the House Finch MG [6] (Figure 2).

In order to compare our rate estimates with the 73 taxon, 1.3kb alignment, we also estimated these quantities using BEAST, again from 8 different initial values, assuming a model of population change and using the HKY+G+I substitution model. This estimated the mean rate as 3.23e-5 (95% HPD 6.37e-6 to 6.239e-5), and the common ancestor of the HF strains and HF strains to have lived 456.7 (95% HPD 130.8 to 969.4) years prior to 2007. We caution that the estimates of the divergence dates from HF to poultry strains are very coarse and should be interpreted with caution, as the modern poultry industry likely alters the population dynamics of MG transmission in ways that may strongly violate the coalescent model assumed in BEAST.
