# Supplementary material for: Ultrafast Evolution and Loss of CRISPRs Following a Host Shift in a Novel Wildlife Pathogen, Mycoplasma gallisepticum
Source: PLoS Genet. 2012 Feb 9;8(2):e1002511. doi: 10.1371/journal.pgen.1002511 (PMC3276549; doi:10.1371/journal.pgen.1002511)
Supplement: Text S3 — Evaluating the effect of frameshift and nonsense mutations. (PDF) [file pgen.1002511.s021.pdf]

Text S3: Evaluating the effect of frameshift and nonsense mutations

We looked for frameshift and nonsense mutations, which we refer to collectively as disrupter mutations. To find these mutations, we *de novo* assembled the 454 reads from our House Finch MG samples, and searched for proteins in the assemblies that had such mutations in them. As the 454 *de novo* assembler improves with increasing read coverage, we restricted our analysis to two of our samples with high sequencing coverage, AL_2007_37 and VA_1994. These strains also bookend the time period of this study. Because the TK_2001 strain is so genetically similar to the MG strains in this study isolated from the House Finches, we also searched assemblies generated from its sequencing data, using the CLC genomics workbench v.3.7.1. For all of these strains, we searched for disrupter mutations present in any of the genes annotated in the reference genome, except for genes with strong similarity to other parts of the genome as these genes are most likely to be misidentified or misassembled. We excluded any gene that was annotated as a VlhA gene or a transposon, or that contained a sequence over 100 bp in length that aligned to another area of the genome with over 85% identity as determined by megablast. By this method 105 of 763 genes (13.7%) were excluded.

For each gene of the remaining 658 genes we used the *de novo* reconstructed gene sequences to check for the presence of disruptor mutations. We considered a gene successfully reconstructed if we were able to find a matching segment amongst the assembled contigs that covered the entire gene (as determined by evaluating local alignments determined by Megablast), and that did not differ by more than 200 bp in size. We were able to find matches for all but 48 of the 658 genes (~93% recovery) in our VA_1994 strain, all but 41 in AL_2007_37 (~94%) and all but 20 (97%) in the TK_2001 strain. 17 of the genes were not recovered in VA_1994 and TK_2001 because they had been deleted along the branch leading from the reference MG strain to our isolates, while such deletions caused 29 genes to be unrecoverable in AL_2007_37. The remaining genes were excluded either because they were not completely covered by a single assembled contig, or in one case because an IS element was inserted into it.

To detect pseudogenizing mutations, each of the 617(AL) , 610 (VA) and 622 (TK) successfully reconstructed genes was translated to detect nonsense or frameshift mutations. This identified 85 possible mutations affecting 76 genes in AL_2007_37 and 99 possible mutations affecting 91 genes in VA_1994. For each of these mutations, we then examined the reads supporting them by evaluating the alignment of the reads to the reference genome in the .ace file produced by both the Newbler and Mosaik aligners. We found that many of the indel mutations were near homopolymers where the underlying reads often both supported and contradicted the presence of the relevant indel mutation. We disregarded all such ambiguous cases unless the reads supporting the presence of the indel outnumbered those contradicting it by 10. This criterion excluded 55 mutations in VA_1994 and 41 mutations in AL_2007_37. This left 44 mutations affecting 42 genes in AL_2007_37 and 44 affecting 43 genes in VA_1994. All of these mutations were shared between VA_1994 and AL_2007_37, except for two. One putative nonsense mutation along the branch leading to AL_2007_37 (reference position: 30,546) was found to have occurred in a gene that had already suffered a frameshift in the common ancestor

of VA_1994 and AL_2007_37. A second mutation was present in VA_1994, but because this area of the genome had been deleted in AL_2007_37, it could not be recovered from this sample.

Of the 45 disruptor mutations found, we excluded an additional 18 mutations because the mutation either occurred in a gene that had been annotated as a pseudogene, or because the mutation was actually supposed to be the wild type state of the gene. The later are likely due to sequencing errors or mutations in the reference genome and we determined this to be the case if the effect of the mutation was to merge two pseudogenes back into a functional protein, and if the mutation was present in all of our sequenced poultry strains as well. This left a total of 27 total disruptor mutations which we grouped into the following two categories. All mutations present in the VA_1994 strain were also present in the closely related TK_2001 strain, and some were present in the other poultry strains.

a) Extension Mutations

4 frameshift mutations had the effect of simply extending the length of the protein shown below. These mutations all occurred within the last 1% along the length of the protein, and although these changes do alter the amino acids towards the end of the protein, it is likely that these proteins remain functional.

Genes with extension mutations

Protein ID	Mutation Location	Mutation	Length of extension (aa)	Present in All Strains?
MGA_0809	132,809	A deleted	5	Yes
MGA_0812	135,135	T->A interrupts stop codon	5	Yes
MGA_1153	416,101	Single T deletion in TK_2001, AL and VA have a deletion of 2 "T"'s at this location	2	Only House Finch MG strains and TK_2001
MGA_0232	718,459	A deleted	11	All but TN_1996

b) Pseudogenes Formers

Excluding the extension mutations and mutations that disrupted the reading frame in one gene but merged it with an upstream coding sequence, we observed 23 mutations affecting 17 genes. These were distributed as 10 insertions, 10 deletions and 3 mutations of an amino acid coding codon to a stop codon. The mutations were often clustered in the same gene. There are 4 genes each of which had 2 mutations which would have disrupted the original reading frame, as well as 1 gene with 3 disruptor mutations. The remaining 12 genes were only disrupted by one mutation. The genes affected by these mutations are given in table S9.
