# Supplementary material for: Ultrafast Evolution and Loss of CRISPRs Following a Host Shift in a Novel Wildlife Pathogen, Mycoplasma gallisepticum
Source: PLoS Genet. 2012 Feb 9;8(2):e1002511. doi: 10.1371/journal.pgen.1002511 (PMC3276549; doi:10.1371/journal.pgen.1002511)
Supplement: Text S4 — Transposon (IS) Movements. (PDF) [file pgen.1002511.s022.pdf]

Text S4: Transposon (IS) Movements

To identify areas of transposon insertion, and to determine if our isolates contained transposable elements in the same location as the reference genome, we developed a method to identify and annotate transposable elements from the 454 reads. Briefly, the method uses a querying strategy similar to BLAST to search for reads that contain sequences identified with the edges of IS elements. The method then annotates the portion of the read that belongs to the IS element, and maps the remaining portion of the read back to the reference genome in order to identify the location where the IS abuts a portion of the genome. The source code for the method is available from the authors upon request.

The reference genome contains members of 2 groups of IS elements. The first group present is identified by the ISFinder database [16] as belonging to the IS1634 family, and is represented in the reference genome by two complete transposases and one shorter fragment with high similarity to a complete IS element (we refer to such fragments as a scar). The second group includes members of the larger IS256 family, and is represented by ten transposases in the genome (although one of these is broken apart by another copy of an IS which has been inserted into it).

The transposases belonging to the first group (IS1634) have also been found in the genomes of other *Mycoplasma* species including *bovis*, *mycoides*, *hyopneumoniae* and *synoviae* [16]. Although this transposase seems to effectively persist in these other *Mycoplasma* genomes, it appears that no functional copy of this transposase remains in this study's House Finch MG strains. Of the two transposases annotated in the reference genome, only one was functional as the other had a frameshift mutation in it. Based on the Newbler assemblies of our sequence data, this particular transposase is even more degraded amongst the strains we sequenced. The first stop codon now appears only 30 amino acids into the gene in all the strains where we could confidently reconstruct it. The only remaining member of the family present in the reference genome, with the only functional transposase, is gone entirely from the House Finch samples we sequenced. It appears that this remaining functional transposase recombined with one of its scars, leading to a large deletion and the destruction of this last functional copy.

In contrast, the second group of transposases, belonging to the IS256 family, has been active during the divergence from the most recent ancestor of our samples and the reference genome. In the reference genome, this group is represented by 10 transposases and 3 small scars. However, in our samples, only 4 of these 10 IS elements are present. Three IS elements in the reference genome had not been inserted by the time the reference strain and the strains in our samples diverged, and three of the other IS elements were located in a region of the genome that had been deleted in the lineage leading to the common ancestor of all of our samples.

Along the branch leading to the common ancestor of all our samples, this element inserted itself into 6 new locations (Table S8). Each of these insertions shown was present in every one of our HF samples, and no sample had any insertion that was not present in the others. Of the 6 IS element insertions, 4 were in intergenic regions, which given the density of genes in the reference genome is highly unlikely ($p < 0.003$). A likely explanation for this bias is that selection is filtering out insertions that destroy functioning genes.
