# Supplementary material for: Ultrafast Evolution and Loss of CRISPRs Following a Host Shift in a Novel Wildlife Pathogen, Mycoplasma gallisepticum
Source: PLoS Genet. 2012 Feb 9;8(2):e1002511. doi: 10.1371/journal.pgen.1002511 (PMC3276549; doi:10.1371/journal.pgen.1002511)
Supplement: Text S5 — Searching for Novel Genes in the House Finch MG isolates. (PDF) [file pgen.1002511.s023.pdf]

Text S5: Searching for Novel Genes in the House Finch MG isolates

This study relied on comparing the assembled genomes of the 12 House Finch MG isolates with that of an annotated reference strain. Given the amount of divergence between the reference and our samples, it was important to determine if using this reference genome would prevent us from analyzing additional gene sequences that were not present in the reference genome but that could provide additional information for this study. To investigate the presence of potentially novel genes in our House Finch isolates, we searched the contigs generated via *de novo* assembly for DNA sequences that could not be mapped back to the reference genome. To do this, we megablasted all of the assembled contigs against the reference genome, and examined any section of a contig sequence longer than 100 bp that could not be mapped to the reference genome. To maximize our chances of detecting any novel sequences in the assembled contigs, we examined the contigs generated from our high-coverage VA_1994 and AL_2007_37 strains. We also pooled all of our 2007 samples for *de novo* assembly and investigated the contigs that were generated from this meta-sample.

Few if any novel DNA sequences were found and arguably none were truly unique because they all had strong similarities to members of either the VlhA or AprE-like proteins present in the reference genome. Of the sequences that failed to align with high similarity to the reference genome, several sequence segments ranging in size from 100bp to 2.1 kb could be identified as similar to a VlhA region by BLAST or BLASTX. However, the largest segment of these that aligned with less than 80% similarity to a portion of the reference genome was only 1.6 kb in size, and a translation of this sequence revealed that it contained a Vlh-A type gene. Similarly, there was a ~500 bp segment that could not be mapped to the reference genome, but this segment was flanked by ~3.3 kb of DNA sequence that had between 66-70% similarity with the other AprE-like proteins present in the genome. These results were consistent for all of the assemblies tested. Given the difficulty in reconstructing these repeat-rich loci and their unsuitability for calling SNPs, we did not pursue these segments further.
