# Supplementary material for: Ultrafast Evolution and Loss of CRISPRs Following a Host Shift in a Novel Wildlife Pathogen, Mycoplasma gallisepticum
Source: PLoS Genet. 2012 Feb 9;8(2):e1002511. doi: 10.1371/journal.pgen.1002511 (PMC3276549; doi:10.1371/journal.pgen.1002511)
Supplement: Text S6 — Detecting recombination. (PDF) [file pgen.1002511.s024.pdf]

Text S6: Detecting recombination

Despite the small amount of genetic variation segregating amongst our House Finch *Mycoplasma* samples (only 412 SNPs), it is not possible to build a single phylogenetic tree with no homoplasies from this data. Similarly, although our poultry strains contained many more SNPs between them, one still cannot infer a single phylogenetic tree that has much more support than alternate trees. The reason for this is not that the SNPs provide very little information about phylogenetic relationships, but rather that many SNPs provide information that is in conflict with the information provided by many other SNPs as determined by the four gamete test. This type of behavior is expected if genes are flowing horizontally as well as vertically in a population, and so we formally tested for the presence of recombination in our dataset.

A plethora of tests are available to detect recombination in sequence data (see ref [7-9]). However, because many of these tests examine the same fundamental signal of recombination, such as the physical clustering of phylogenetically concordant SNPs, they commonly yield qualitatively similar results when performed on the same dataset. To detect recombination in our combined House Finch and poultry strain dataset, we used the pairwise homoplasy index test[7] as implement in splitstree4 [10]. Examining the entire data set, this test found a statistically significant signal of recombination (p < 1e-9). This signal comes predominantly from the four newly sequenced poultry strains because there is not enough genetic variation to make the test significant when only the house finch strains are considered. However if we apply to the house finch MG strains the homoplasy test by Maynard-Smith and Smith [11], which is found to perform well in situations of low nucleotide diversity [9], we still obtain a significant signal for recombination. This test differs from the pairwise homoplasy index test, in that rather than looking for spatial clustering of phylogenetically concordant SNPs, it instead asks if the number of homoplasies observed on a tree is particularly large given the number of mutations on the tree and the number of sites that were available to mutate. To implement this test, using the dnapars program in the phylip package we first found a parsimonious tree for the House Finch isolates while including the reference genome as an outgroup, and then counted the number of homoplasies that appear only within the clade of House Finch MG isolates. This identified 13 homoplastic mutations out of a total of 412 variable sites in an alignment of approximately 756.5 kb of DNA. Intuitively, it seems extremely unlikely to see so many homoplastic mutations given the large number of sites that were available to mutate. However, exactly quantifying how unlikely this is complicated because different sites in the genome evolve at different rates, so that homoplasies are much more likely to appear at some sites than others. To get around this issue, the original paper describing the test proposed reducing the total number of sites in the alignment down to a smaller number of effective sites. This paper described a heuristic method to estimate how much one should reduce the alignment size, but this method required a sequence from a distant outgroup that fulfilled a difficult set of assumptions. In practice, since having such an outgroup and trusting that it satisfies the assumptions is rare, many researchers simply take the effective number of sites to be equal to 0.6 multiplied by the total number of sites in the alignment. This 0.6 value was selected as a conservative choice when it was first used in a paper comparing different methods of testing for recombination [9] because it was much less than the inferred values in the original paper (0.73-0.83) and because 0.6 was given as the lower limit for a believable estimate in that same paper during a discussion of different methods to estimate the effective number of sites. Since then, this 0.6 value has been used widely in other papers and is

the default setting implemented in software packages that implement tests for recombination such as START (Sequence Type Analysis and Recombinational Tests, [12]). Suffice is to say, it is clear that there is some ambiguity in how best to determine the effective number of sites that are available to mutate, particularly when there is not complete sequencing for every strain, and as a result the homoplasy test could be considered overly conservative or subjective depending on one's prior beliefs. However, because the observed number of homoplasies in our dataset is so unlikely, we can confirm that it is extremely unlikely even given a wildly conservative set of assumptions. To determine the effective number of sites we used in our test, we first dropped the total number of sites in the alignment from 756,552 bp, down to the total number of sites where all the strains had data present which was only 273, 482 bp. This is obviously an overly conservative reduction as the vast majority of sites in the alignment had data for a majority of strains. Next, instead of applying the standard 0.6 correction to this reduced number, we applied a much more stringent criterion of 0.2, leaving us with 0.2 x 273482 = 54,695 effective sites, or only 7% of the original alignment length. We then estimated the probability of observing 13 or more homoplasies by simulation. Of 1 million simulations, the highest observed number of homoplasies was only 9, and we thus estimated our p-value as $p<1e\text{-}6$. However, the probability of observing so many homoplasies is almost certainly lower than this bound, not only because every assumption we made is expected to increase the p-value, but also because in our dataset two sites needed to convergently mutate not twice, but three times each in order for them to be in agreement with the tree. Since the homoplasy test treats all homoplasy counts as equivalent, even though repeated homoplasies at the same site are particularly unlikely, this again introduces a conservative bias into the test. We also note that the homoplasy test does not consider that a mutation at a site need not produce the exact same basepair each time, as there are three basepairs available to mutate to, which introduces yet one more conservative bias into the test.

Having established that *Mycoplasma gallisepticum* is a bacterium that recombines, we next sought to characterize the nature of recombination in this organism. To bookend a continuum with a dichotomy, recombination between microorganisms can be described as either chunky or smooth. Recombination is chunky when the recombination rate is much lower than the mutation rate, so that the genome is filled with large blocks of easily identifiable DNA that have a shared history that is in strong disagreement with the phylogenetic pattern exhibited by other sections of the genome. In contrast, recombination is smooth when the recombination rate is nearly equal to or greater than the mutation rate, in which case clusters of phylogenetically concordant SNPs tend to be much smaller and correctly delineating a specific section of DNA that has not recombined since the last common ancestor is impossible to do with any reasonable certainty. We therefore looked at the size distribution of phylogenetically concordant chunks to examine the extent to which the statistically significant finding of recombination was due to a few large blocks, or many smaller blocks.

To do this, we systematically determined the size distribution of phylogenetically concordant genomic segments in our sequenced isolates by implementing a recursive method that assigned each possible basepair in the genome to a phylogenetically concordant segment. Our method, illustrated in Fig S3**,** proceeds as follows. First for the strains under study we enumerate all possible unrooted trees. Next, for each phylogenetically informative SNP in the genome, we determine which trees are compatible with and incompatible with the pattern of variation shown at that SNP. In the next step, for each tree we determine all blocks in the genome that are in

agreement with that tree by assigning regions of the genome with consecutive compatible SNPs to single continuous block, and allowing half of the genome between a concordant SNP and a disconcordant SNP to be included in the block. Finally, all trees are examined to determine which has the largest block, this block is assigned to the tree, then the segments in each tree are updated to account for this, and this is repeated until every position in the genome is assigned to a block.

To implement the recursive method on our dataset, we first disregarded the data from the House Finch MG isolates. The House Finch MG isolates have too little genetic variation to usefully determine spatial patterns of recombination and were nearly genetically identical to the TK_2001 poultry isolate. By only using the MG poultry isolates and the reference genome, it is possible to work with the full enumeration of possible unrooted trees as there are only 15 and so we could avoid approximate and heuristic methods. The distribution of sizes of phylogenetically concordant blocks is shown in Fig S4, which also displays a distribution obtained by randomly rearranging the patterns of genetic variation shown at each SNP to different positions in the genome.

Fig S4 shows that the signal of recombination in our dataset is not due to a few rare transfer events, but that these genomes are reasonably mixed, as there are a large number of sizable concordant blocks that are in agreement with different trees. We note that we can also test for recombination by creating permutated datasets that keep the position of SNPs fixed and randomly reassigning the patterns of genetic variation shown at each SNP. If spatial clustering is significant, then the number of blocks required to assign the entire genome to a segment should be much less than the number required in a permutated dataset. Fig S5 shows the distribution of blocks required in 2,600 random permutated datasets, and as expected the total number of blocks required is much greater than that required in the actual dataset, again indicating that recombination is statistically significant with a vanishingly small p-value.
