# Supplementary material for: Ultrafast Evolution and Loss of CRISPRs Following a Host Shift in a Novel Wildlife Pathogen, Mycoplasma gallisepticum
Source: PLoS Genet. 2012 Feb 9;8(2):e1002511. doi: 10.1371/journal.pgen.1002511 (PMC3276549; doi:10.1371/journal.pgen.1002511)
Supplement: Text S7 — Effect of recombination on the estimated substitution rate and demonstration of true temporal signal. (PDF) [file pgen.1002511.s025.pdf]

Text S7: Effect of recombination on the estimated substitution rate and demonstration of true temporal signal

The presence of recombination could bias our estimate of the substitution rate as inferred from BEAST. The MCMC algorithm used within BEAST proposes and evaluates the parameters in an evolutionary model based on a single phylogenetic tree. However, in the presence of recombination, there is no single phylogenetic tree that represents the history of all of the genomes sequenced, and this discrepancy between the biological reality and the inference model could affect our results. Although we hope future computing developments that use the ancestral recombination graph approach will eventually solve this problem by allowing the current Bayesian inference approaches to account for recombination, at present there are no available methods to systematically perform simultaneous inference of the posterior distributions for all the evolutionary parameters in circular-genome datasets as large as ours. However, despite this difficulty, it is clear that the single best point estimate for the mutation rate will always be on the order of 10^{-5} per site per year, and that given a number of well supported assumptions, that the interval of uncertainty around this estimate will encapsulate this rate to within an order of magnitude.

To demonstrate that our conclusions are robust to the presence of recombination, we note that a simple method of inference which is unaffected by recombination gives virtually identical results. A naïve estimate of the mutation rate can be obtained for any two pair of sequences simply by dividing the number of mutations that appear between the earlier sample and the later sample by the amount of time separating the two samples. Although the expected value of this estimate is unaffected by recombination, this naïve estimate is biased towards a higher rate because it does not account for the time between the last common ancestor of the two samples and the initial sampling of a sample, which is additional time during which mutations could appear. However, the nature of our data is such that this bias is very small, and any realistic correction for this bias does not substantially change the inference. The reason for this is that the most common ancestor of all of the House Finch MG strains was almost certainly present near the time of our initial sampling period, as supported by three lines of evidence. First the epizoodic was very well documented as beginning in 1994 by a wide variety of observers, and despite ample opportunity there were no reports of MG infection in House Finches before this date. Second, and in agreement with 1994 being the first year when MG infected House Finches, in a broad sampling of MG from a variety of host species, all of the House Finch MG strains were genetically identical, despite a large amount of diversity in the poultry population, indicating a recent founder event (Fig S1). Finally, our genome level sequencing of the 1994 strains provides additional evidence for this interpretation. The 1994-1995 samples are characterized almost exclusively by singletons (Table S2), indicating a recent common ancestor and population expansion, and therefore a small bias in the naïve estimate. Therefore, given that there was a bottleneck in the founding of the house finch MG strains, the excess time not accounted for by the difference in sampling times is expected to be very small, on the order of a few months compared to the 13 year interval between the 1994 and 2007 samples, meaning that this naïve method, equivalent to a Poisson regression, will provide a very good estimate of the substitution rate. Evaluating this naïve estimate over any given pairwise comparison of 1994 and 2007 strains we get an estimated rate of 1.35-2.36 X 10^{-5} with an average of 1.7 X 10^{-5}.

Although calculating an interval of uncertainty around these estimates is dependent on assumptions about the evolutionary process, one assumption that is uninfluenced by the effects of recombination is to assume that mutations are introduced into the genome as a constant Poisson process. With this assumption, the lower interval for the 95% confidence interval of our mutation rate is still on the order of 10^{-5} for the strains in this study. Although violations of a constant Poisson process are some of the most frequent findings in the field of molecular evolution, correctly identifying and modeling such deviations would require much broader sampling of bacteria then this study, or any other published study we are aware of, could provide. However, all indications are that such violations are not large in magnitude (Fig 4), and even if the width of the 95% confidence interval for the rate estimate assuming a Poisson process is doubled in size, the lower bound of the confidence interval is still approximately 10^{-5}. Therefore, we find no plausible violations of the model large enough to substantially alter our rate estimate more than an order of magnitude.

Additionally, as a simple test and demonstration that our data do contain a true temporal signal and the estimated rate is also not an artifact of the BEAST analysis, we used the program Path-O-Gen to evaluate the clock like nature of the data. An ML tree without an assumed clock was first estimated using the program PhyML [13] and the HKY substitution model used in our BEAST analysis. The regression in Path-O-Gen obtained an estimated rate of 1.45×10^{-5} using the default root for the tree ($R^2 = .68$) and it estimated a rate of 9.6×10^{-6} ($R^2 =.92$) using the best-fitting root, confirming that our Poisson regression results and the BEAST analysis are in agreement with this separate method of estimation. Finally, we also performed a randomization test as described in [14] by randomly reassigning the dates of all of our House Finch strains and rerunning our BEAST analysis We performed this randomization 20 times and each time obtained and HPD interval for the rate that did not overlap with our current estimate and was below our current estimated interval.
