# Supplementary material for: Ultrafast Evolution and Loss of CRISPRs Following a Host Shift in a Novel Wildlife Pathogen, Mycoplasma gallisepticum
Source: PLoS Genet. 2012 Feb 9;8(2):e1002511. doi: 10.1371/journal.pgen.1002511 (PMC3276549; doi:10.1371/journal.pgen.1002511)
Supplement: Text S8 — CRISPR Analysis (PDF) [file pgen.1002511.s026.pdf]

Text S8: CRISPR Analysis

To annotate the CRISPR elements in our 454 data and Illumina data we designed the program that computationally reconstructs a CRISPR locus from the sequence data, and simultaneously provides visualization tools that allow the user to validate the computational reconstruction, detect polymorphism, and manually check any ambiguities that may appear during the reconstruction. Each read generated from a sample is checked to see if it contains a sequence similar to the CRISPR repeat present in the ancestral genome. Any reads that contain such a sequence are selected into a subset of reads for further inspection. For each read in this subset, a dynamic alignment algorithm is used to identify the portions of the read that belong to the CRISPR repeats, and by exclusion, those portions that belong to the CRISPR spacers. Spacers that are exactly the same, or that appear to only differ due to sequencing errors, are then identified as spacer families, and these families are each given a numeric name determined by their (essentially random) order of discovery. Finally, as in many modern genome assemblers, the complete CRISPR locus is reconstructed through means of a graph. Each spacer family represents a node that can be placed either upstream or downstream of other families that appear in the same read as themselves. The program constructs this graph, determines the order of the observed spacer families, and plots it in simple format for the user.

We used this method to reconstruct the complete CRISPR locus in all 16 of our samples (Table S12). Of the 61 unique CRISPR spacer regions present in the reference genome, none are present in our samples, which collectively have gained a net total of 47 unique spacers since the time of their divergence from the reference genome. Table S8 shows the number of CRISPR spacers in each strain. Our GA_1995 sample has a copy of every spacer found in every other HF MG strain as well as TK_2001, and thus all other House Finch MG genotypes can be represented by deleting or duplicating the CRISPR spacers found in this strain. As such, the CRISPR array in each strain can be represented as a vector of discrete character states. Because adjacent CRISPR spacers are likely to be lost by the same deletion events, we reconstructed the CRISPR tree (Fig 5) using a parsimony method that always allowed deletions of neighboring CRISPR regions to be scored as single events. Following this assumption, we grouped strains into clades based on the presence of shared deletions or duplications. In instances where two or more equally parsimonious explanations could be provided for a pattern of deletions, we represented this ambiguity in the tree (for example the pattern of deletions shared by 2001 and 2007 can be equally well explained by having or not having these groups share a common ancestor to the exclusion of other strains).

Like the SNP phylogeny, the CRISPR phylogeny is consistent with a single origin of the epizootic and implies periodic replacement of the standing genetic variation in successive cohorts (2001, 2007). Although the deletions and expansions of most of the CRISPR spacers shown are likely due to strand slippage or recombination between the CRISPR repeats, the loss of the CRISPR spacers at the start of the locus in the 2007 strains is part of a much larger deletion of 12.7 kb that is unique to these strains and involves an alternative mechanism (deletion 4 in Table S7).

A recent investigation of CRISPR spacer repeats in *Yersinia pestis* found that a majority of spacers found in the CRISPR array originated from other areas of the organisms genome,

indicating that the CRISPR loci might be involved in regulating intra-genome dynamics, such as controlling gene expression levels or IS/prophage proliferation[15]. To determine if this was also true for the spacers we observed in *Mycoplasma gallisepticum,* we blasted each of the 302 unique spacer sequences that we found against the reference genome (blastn, with parameters "-*W 7, -e 1, -F F -r 2"*), and looked for any sequences that had an alignment score over 40 (equivalent to a ~66% match) to the reference genome. This analysis showed that 3 of the 302 spacer sequences were perfect matches to other portions of the genome, meaning they likely originated from proto-spacers within the MG genome. One spacer found within the reference genome was derived from a sequence within a hypothetical membrane protein (annotated MGA_0908), and another from the reference genome was a perfect match to a segment of DNA topoisomerase IV subunit A (MGA_0056). The third perfect match was from the CK_1996 strain, which contained a spacer sequence derived from a VlhA.4.01 lipoprotein gene (MGAH_0966).

To determine if any of the other CRISPR spacers were similar to any previously sequenced organisms, we blasted each of the 302 spacers against the NCBI 'nr' blast database and examined the top hit after excluding any hits to MG genomes. The top scoring hit only had a score of 50, and the top 5 hits were to *Schistosoma mansonis,* Human*,* and Zebrafish, leading us to conclude that there we no significant matches. Based on these comparisons to known DNA within and outside of the MG genome, we concluded that the source of the CRISPR spacers in this study is predominantly was from previously unstudied organisms.
