# Supplementary figures and images for: Correction: Ultrafast Evolution and Loss of CRISPRs Following a Host Shift in a Novel Wildlife Pathogen, Mycoplasma gallisepticum
Source: PLoS Genet. 2012 Mar 5;8(3):10.1371/annotation/b5608bc6-aa54-40a7-b246-51fa7bc4a9db. doi: 10.1371/annotation/b5608bc6-aa54-40a7-b246-51fa7bc4a9db (PMC3293929; doi:10.1371/annotation/b5608bc6-aa54-40a7-b246-51fa7bc4a9db)

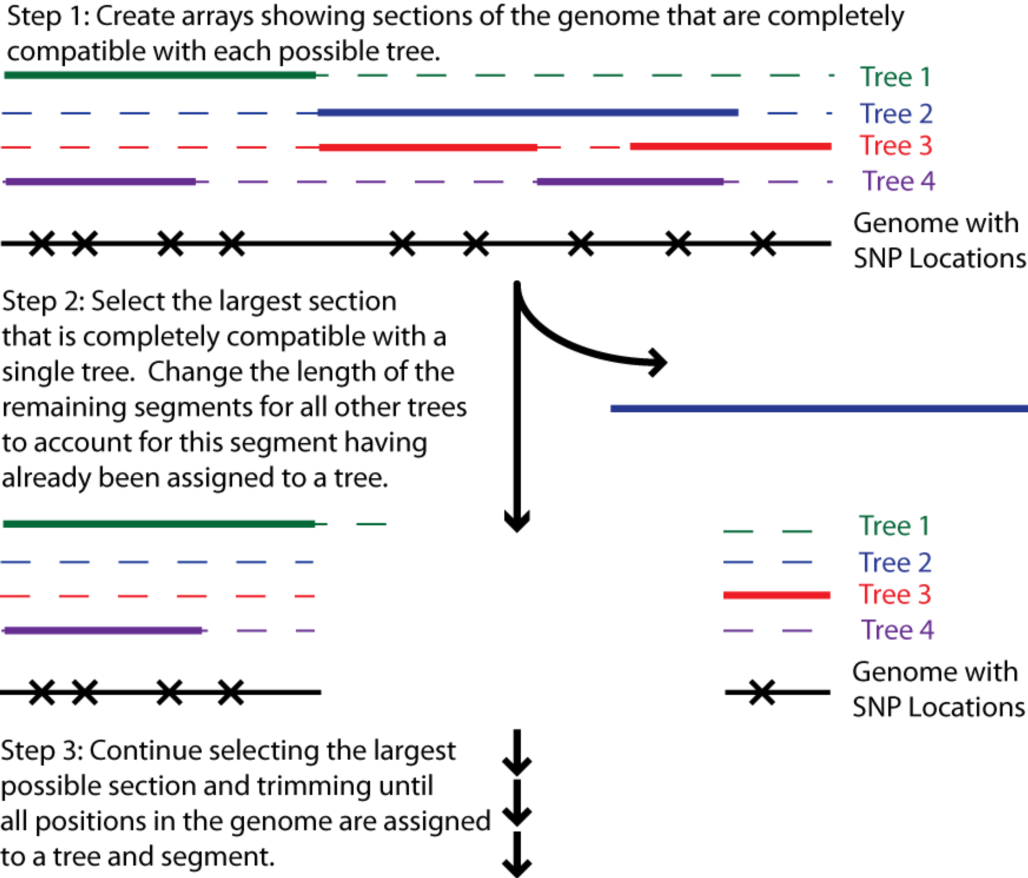

Supplement: Supplementary file 1 [file pgen.b5608bc6-aa54-40a7-b246-51fa7bc4a9db.s001.tif]
